# LEADD: Lamarckian evolutionary algorithm for de novo drug design

**DOI:** 10.1186/s13321-022-00582-y

**Published:** 2022-01-15

**Authors:** Alan Kerstjens, Hans De Winter

**Affiliations:** Department of Pharmaceutical Sciences, Faculty of Pharmaceutical, Biomedical and Veterinary Sciences, University of Antwerp, Universiteitsplein 1A, 2610 Wilrijk, Belgium

**Keywords:** De novo drug design, Evolutionary algorithm, Synthetic accessibility, Fragment-based, Graph-based

## Abstract

Given an objective function that predicts key properties of a molecule, goal-directed de novo molecular design is a useful tool to identify molecules that maximize or minimize said objective function. Nonetheless, a common drawback of these methods is that they tend to design synthetically unfeasible molecules. In this paper we describe a Lamarckian evolutionary algorithm for de novo drug design (LEADD). LEADD attempts to strike a balance between optimization power, synthetic accessibility of designed molecules and computational efficiency. To increase the likelihood of designing synthetically accessible molecules, LEADD represents molecules as graphs of molecular fragments, and limits the bonds that can be formed between them through knowledge-based pairwise atom type compatibility rules. A reference library of drug-like molecules is used to extract fragments, fragment preferences and compatibility rules. A novel set of genetic operators that enforce these rules in a computationally efficient manner is presented. To sample chemical space more efficiently we also explore a Lamarckian evolutionary mechanism that adapts the reproductive behavior of molecules. LEADD has been compared to both standard virtual screening and a comparable evolutionary algorithm using a standardized benchmark suite and was shown to be able to identify fitter molecules more efficiently. Moreover, the designed molecules are predicted to be easier to synthesize than those designed by other evolutionary algorithms.

**Supplementary Information:**

The online version contains supplementary material available at 10.1186/s13321-022-00582-y.

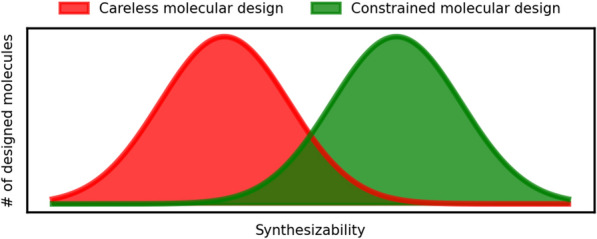

## Introduction

Many computational drug discovery projects employ virtual objective functions (also termed fitness or scoring functions) to predict a molecule’s properties of interest, including its biological activity. In virtual screening (VS) one evaluates the objective function for all molecules in a virtual library to find the most promising ones. Commonly the molecules being screened are either commercially available [1] or predicted to be easy to synthesize [2, 3], enabling a fast transition from in silico to in vitro studies.

However, given that even the largest virtual libraries [2, 4] dwarf in size compared to drug-like chemical space, which is commonly cited to contain somewhere between 10^23^ and 10^60^ [5–7], it’s unlikely that the library will contain the most active molecules possible. Preferences for certain chemotypes and synthetic reactions [8, 9] often make their way to virtual libraries, leading to a small and non-uniform coverage of chemical space [10, 11]. This, coupled to the fact that publicly available libraries may have been screened previously or even contain patent-protected molecules, raises concerns about a lack of chemical novelty. Last but not least, the enumeration, storage, maintenance and screening of virtual libraries is a resource intensive process.

Computational de novo drug design (DNDD), that is, the computational design of molecules without a problem-relevant molecular starting point, has the potential to solve some of these problems. The traditional approach to DNDD [12], sometimes termed goal-directed design [13], is the progressive construction or modification of a molecule to optimize the value of a fitness function, according to some optimization algorithm. While this approach can succeed at finding highly fit molecules efficiently, if applied naively the designed molecules tend to be hard to synthesize [13, 14]. Different solutions have been applied to tackle this problem.

Some solutions revolve around the use of synthetic accessibility (SA) metrics. These metrics may have to be calculated many times throughout the design process, often limiting the user to rather crude rules [15, 16] or heuristics [17–19] and precluding the use of more reliable retrosynthetic analyses [20, 21]. Post-hoc filtering [22, 23], while simple and modular, is computationally inefficient as it might discard solutions in which significant amounts of costs were already sunk. Employing the SA descriptors as a heuristic score bias instead [14, 23] can partially solve this problem. However, both approaches create hard or soft boundaries within the search space respectively. Given that the fitness landscape of a typical drug discovery project can be very rugged, this may impede the discovery of good solutions. Alternatively, one may attempt to optimize both the fitness and SA simultaneously with multi-objective optimization algorithms [24, 25]. Since these objectives may counteract each other, the algorithm attempts to find suitable compromises between them, but it’s the decision-maker’s responsibility to define which balances are desired or acceptable.

A second group of approaches attempts to incorporate some chemical awareness into the design algorithm itself. Ways of achieving this include fragmentation/recombination rules [26, 27] and simulating virtual chemical reactions [28–30]. These have the advantage of considering SA implicitly as part of the molecular construction process. The likelihood of such an algorithm succeeding at designing SA molecules depends on how well it captures chemical reality. Typically, the better a construction scheme resembles organic synthesis, the higher the SA of the designed molecules, but also the computational cost to find them. Reducing the number of bonds created by the algorithm, for instance by using predefined multi-atomic molecular fragments, can be an effective way of increasing the SA of designed molecules while avoiding expensive construction schemes.

More recently, generative models such as variational autoencoders [31], recurrent neural networks [32] and generative adversarial networks [33] have been applied to DNDD. When trained on datasets of molecules with some desirable properties, including synthesizability, these models can suggest new molecules with similar properties. These technologies have shown great promise in designing synthetically feasible molecules [14]. However, the amount of available training data can hamper the approach.

In this paper we describe LEADD, an evolutionary algorithm (EA) for de novo drug design and optimization. EAs have a rich history of being applied to DNDD [16, 24–27, 30, 34–39]. They draw inspiration from Darwinian evolution and natural selection, stochastically breeding a population of solutions through the use of genetic operators (i.e. mutation and crossover). Over the course of generations, the objective function exerts selective pressure on the population driving it towards optimality.

LEADD designs molecules as combinations of molecular fragments, bonded according to the topology of a graph. Knowledge-based atom pair compatibility rules, defining which fragments can be bonded and how, are enforced by a novel set of genetic operators. Both the fragments and compatibility rules are extracted from a library of drug-like molecules, and the outcomes of the genetic operations are biased according to the frequency of the fragments in drug-like matter. Additionally, a Lamarckian evolutionary mechanism adjusts the future reproductive behavior of molecules based on the outcome of previous generations. LEADD attempts to strike a balance between optimization power (OP), SA of designed molecules and computational performance.

## Methods

### Fragment library creation

A virtual library, assumed to be representative of drug-like chemical space, is fragmented to yield the fragments employed by LEADD during the design process.

Within this context, a fragment is a connectivity-encoding molecular subgraph of the source molecule from which it was extracted. A connection is an object describing the bond between two atoms and is directional by nature. It can be represented as a three-integer tuple, where the integers describe the starting atom type, ending atom type and bond type respectively. Bonds are classified into either single, double or triple bond type (aromatic bond types don’t occur since rings aren’t fragmented; see below). While any atom typing scheme may be used, we have implemented into LEADD MMFF94 [40] and Morgan atom types. Morgan atom types derive their name from the Morgan algorithm [41], a variant of which is used in ECFP [42] and RDKit Morgan fingerprints [43] to generate canonical atomic identifiers. These atomic identifiers are 32-bit integers describing the atom’s topological circular chemical environment of a given radius *r*. Said integer is taken as the atom’s Morgan atom type. For clarity, the examples and figures in this paper use MMFF94 atom types.

**Fig. 1:**
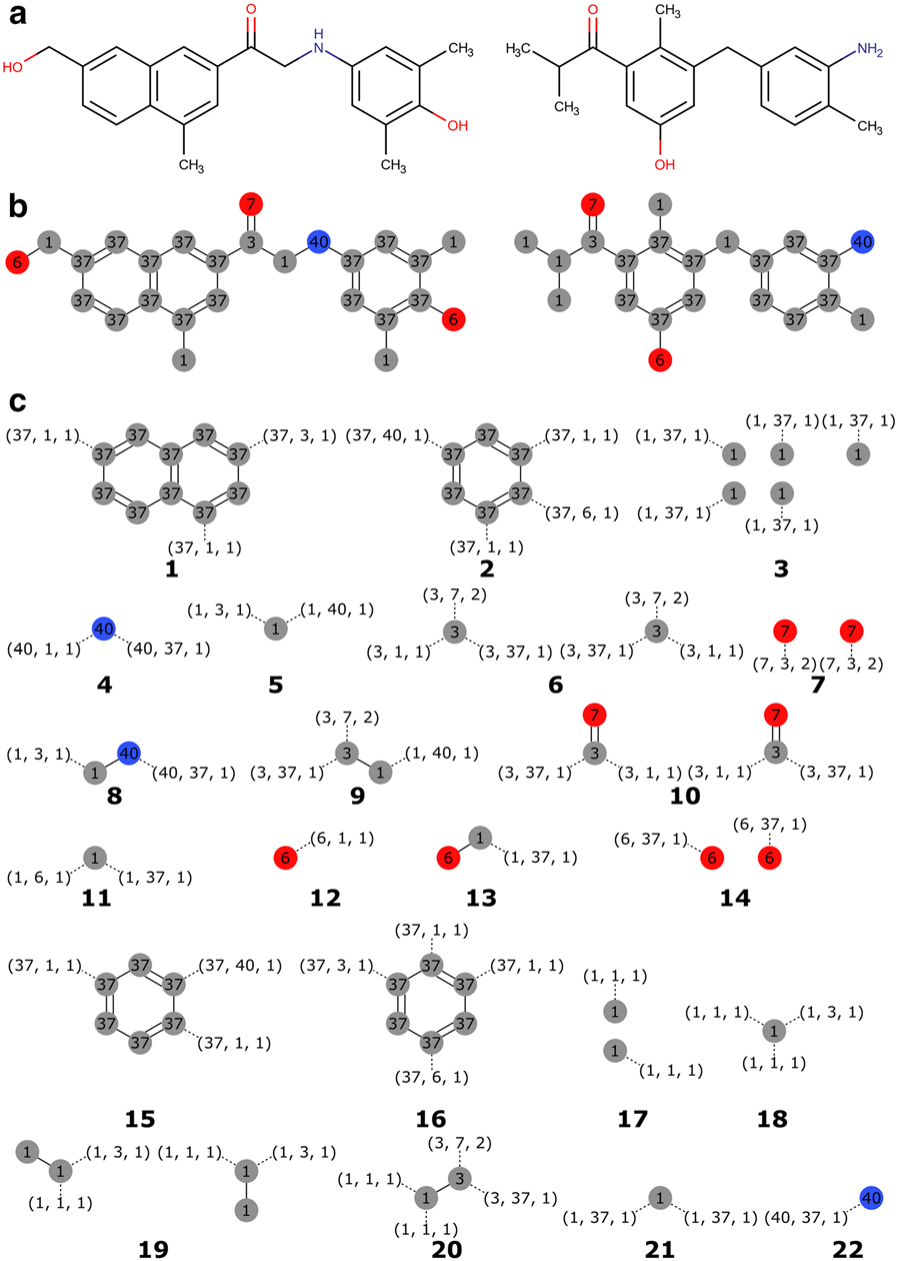
Fragmentation example of two molecules. The input molecules (**A**) are assigned MMFF94 atom types (**B**). Ring systems and all possible subgraphs from the remaining linkers and side chains of a given size (in this example s ϵ [0 .. 1]) are extracted as fragments (**C**). The bonds that were cut to extract fragments become connectors, and are represented as three-membered tuples in parenthesis. The number in bold below each fragment is its ID

We distinguish between connections, which are generic objects describing the type of an atom–atom bond, and connectors, which are specific instances of a connection centered on a fragment’s atom. During molecule fragmentation, the bonds between the fragment’s molecular subgraph and its extra-fragment adjacent atoms are recorded as connectors (Fig. 1).

For each molecule, fragmentation starts by isolating ring systems from the acyclic regions. Rings pertaining to the Smallest Set of Smallest Rings (SSSR) [44] are considered to be part of the same ring system if they share at least one atom. Given the complexities of designing drug-like ring systems, we decided to consider whole ring systems as fragments. The remaining acyclic structures may either be taken as fragments as a whole or subjected to systematic fragmentation by extracting all possible molecular subgraphs of a given size from them, with each subgraph becoming a fragment (Fig. 1). Hydrogens are treated implicitly. The size of the extracted subgraphs (*s*), given in number of bonds within the subgraph, is provided by the user. When *s* = *0*, single atom fragments are generated. Fragments of different sizes can be combined by specifying a range of sizes.

Two fragments are considered equivalent only if both their molecular graph and connectors are the same. Both attributes are encoded as canonical ChemAxon extended SMILES (CXSMILES) [45] and molecular identity is assessed as canonical CXSMILES identity.

The generated fragments, their connectors, frequencies, sizes and other convenience information are stored in a relational SQLite3 database [46] (Additional file 1: Fig. S1). When a generated fragment is already present in the database its frequency is incremented by one.

### Connection compatibility rules

Fragment compatibility is defined at the connection level. Two fragments can be bonded together if two of their free connectors are compatible. Whether two connections are compatible is determined by a set of pairwise and symmetric compatibility rules.

**Fig. 2:**
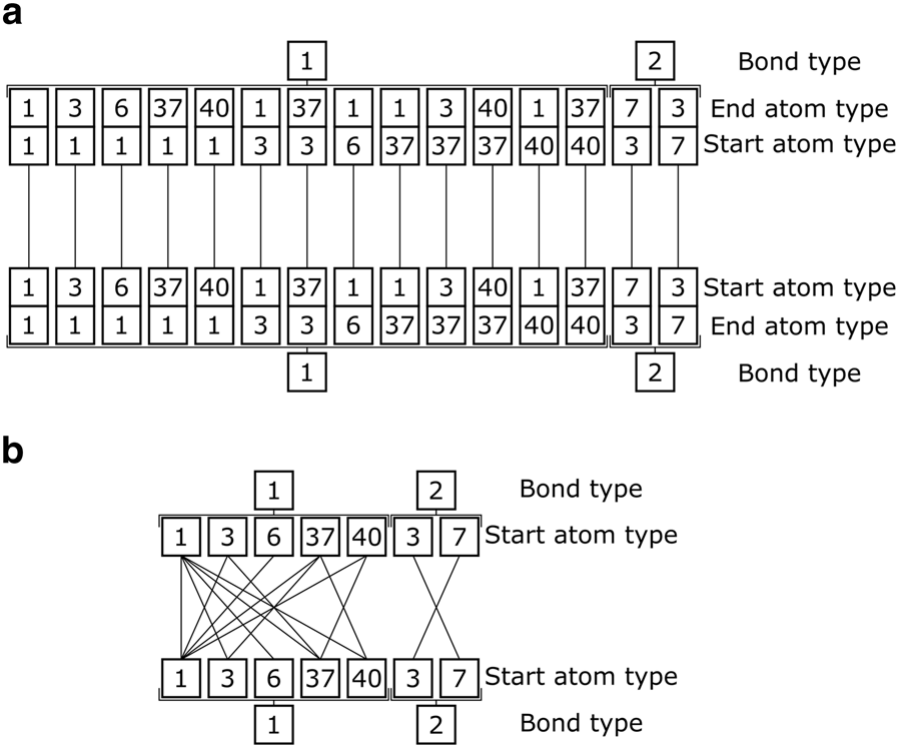
Connection compatibilities of the connections in Fig. 1 according to the strict (**A**) and lax (**B**) compatibility definitions. Since in the lax definition the end atom type is irrelevant it is omitted

The compatibility rules are extracted from the connections table of the fragment database according to a user-specified compatibility definition. We employ two of those definitions, termed the “strict” and “lax” compatibility definitions. Both definitions are illustrated in Fig. 2.

According to the strict definition two connections are compatible only if (a) their bond types are the same, and (b) their atom types are mirrored (i.e. the start atom type of one is the end atom type of the other and vice versa). Consequently, only a single connection is compatible with each connection. During molecule design this entails that the connectivity of fragments to their flanking atoms in their source molecules is preserved. In other words, a fragment must be connected to atoms of the same atom type as those that flanked the fragment in the source molecule.

When following the lax compatibility definition two connections are compatible if (a) their bond types are the same, and (b) if the starting atom type of one has been previously observed paired with the starting atom type of the other in any connection. This definition expands the connectivity scope from the fragment’s source molecule to the entire source molecules pool. In other words, two atom types can be connected if they have been observed paired together in any of the database’s connections, which means they were bonded in at least one of the source molecules. As such, the strict compatibility definition is a subset of its lax counterpart.

### Chromosomal representation and initialization

**Fig. 3:**
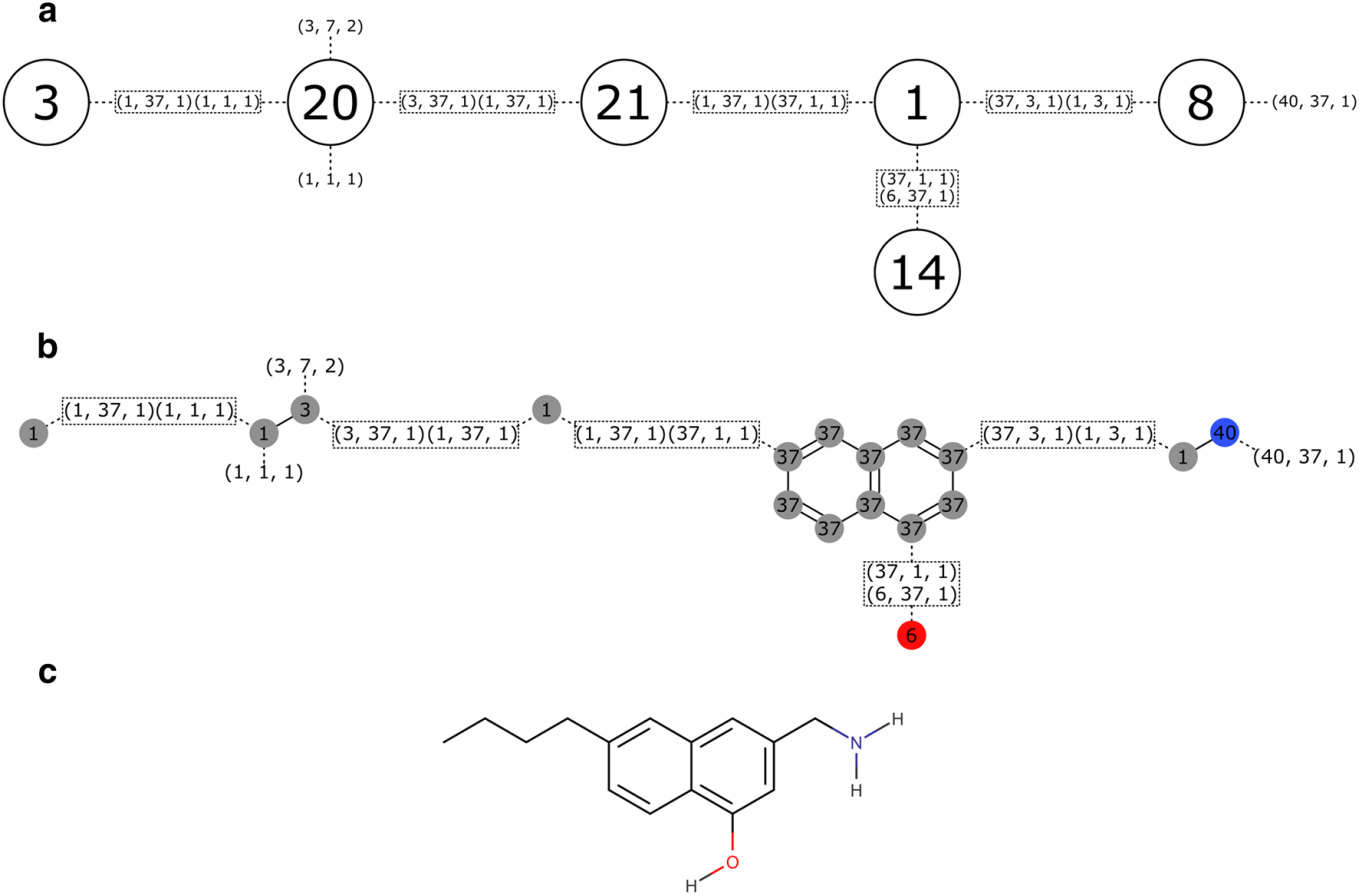
Chromosomal representation of a molecule created through combination of fragments in Fig. 1 using the lax compatibility definition. **a** Chromosomal meta-graph. Numbered vertices correspond to fragment IDs. Numbers between parenthesis represent connector tuples. Bonds between connectors are represented as rectangles. **b** The chromosome with fragments shown as their molecular graphs. **c** Translation of the chromosome to the molecule seen by the user

Molecules are represented internally as meta-graphs [38], where each vertex is a molecular graph corresponding to a fragment, and the edges describe which connectors bind the fragments (Fig. 3). Due to the complexities of designing drug-like ring systems we treat ring systems as whole fragments, represented as a single vertex in the meta-graph. However, while the genetic operators don’t create cycles in the meta-graph, they would work on existing cycles if one were to add a cyclization operator in the future.

The meta-graph chromosome can be translated into a single molecular graph by connecting the molecular graphs of all fragments (Fig. 3). Thereafter, hydrogens are added to satisfy all incomplete valences. For elements with more than one valid valence like sulphur or phosphorus hydrogens are added up to the closest valid valence.

Upon initialization, for true de novo drug design random chromosomes are generated by successively combining random fragments. However, in some instances the user may want to perform molecule optimization instead, starting from a known population of molecules. In this case, it’s possible to convert regular molecular graphs into meta-graphs by following the previously laid out fragmentation procedure using single atom acyclic fragments (*s* = *0*). If any of the connections generated during the fragmentation of starting molecules don't appear in the database, connection compatibility information won't be available for them and the molecule will therefore be skipped.

### Genetic operators

**Fig. 4:**
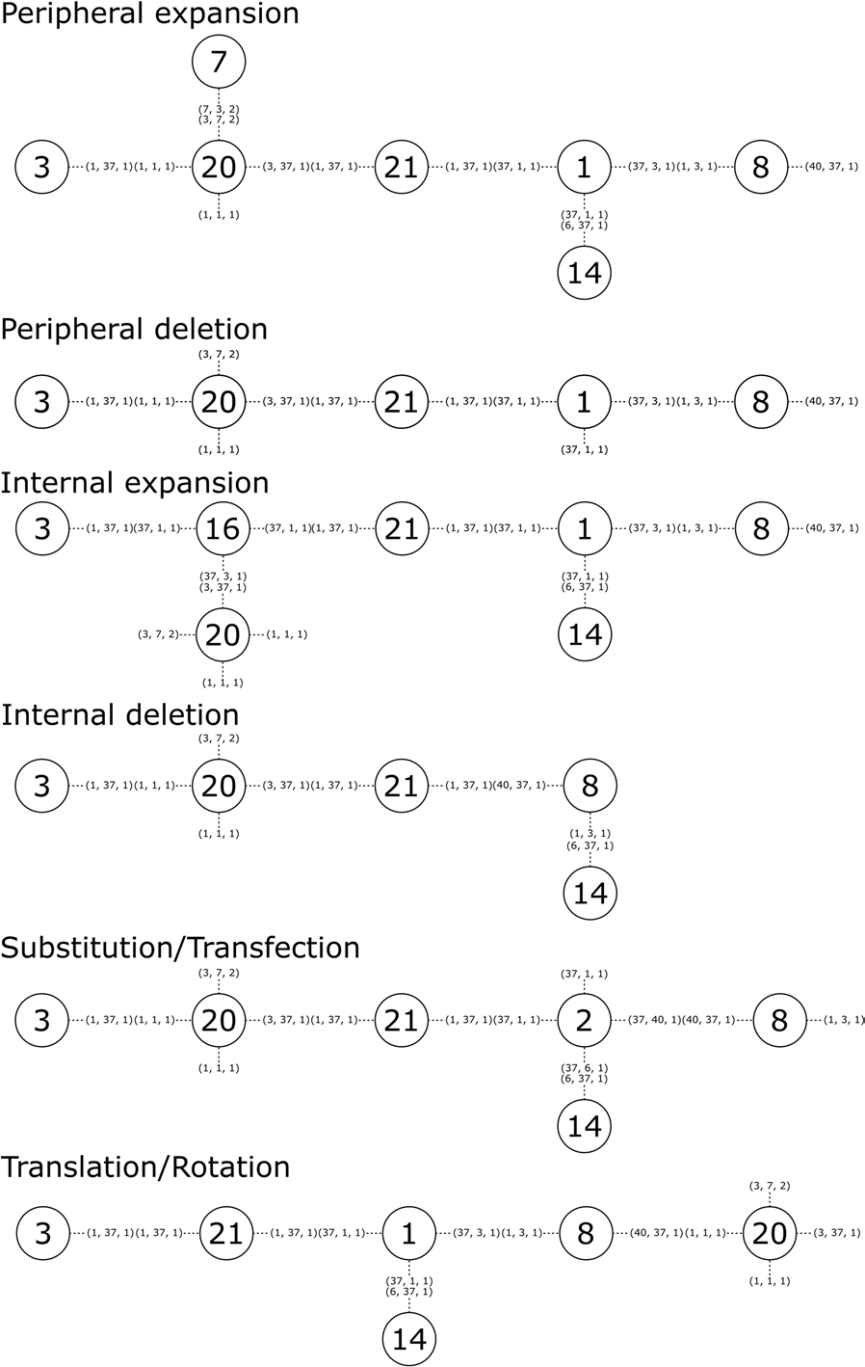
Illustration of the resulting chromosomes after applying each of the eight genetic operators to the chromosome given in Fig. 3a

LEADD employs eight distinct genetic operators to modify the chromosome and generate offspring (Fig. 4). Some of these operators have a peripheral and internal variant, referring to the location of fragments on which they operate. Peripheral fragments are those connected to one or less other fragments (vertex degree *d* ≤ *1*), while internal fragments are those connected to two or more fragments (*d* ≥ *2*). While peripheral operators are theoretically sufficient to access the entirety of the search space, in practice this relies on statistically unlikely sequences of operations, since to modify the core of the molecule one would have to “backtrack” and remove all peripheral fragments obstructing it. Hence, the algorithm would be very likely to get stuck in local minima on the fitness landscape.

The function of peripheral variants is mostly self-explanatory: peripheral expansions attach a fragment sampled from the database to a free connector, while peripheral deletions delete a peripheral fragment.

In internal expansions a fragment is inserted between a target fragment and one or more of its adjacent fragments. For this purpose, connectors involved in bonding the target fragment to the adjacent fragments are considered free.

In an internal deletion an internal target fragment is deleted. This is only possible if one of the fragments adjacent to the target fragment can “take its place” and bond to the remainder of the adjacent fragments.

In a substitution a target fragment is replaced by a fragment in the database. Connectors bonding the target fragment to its neighboring fragments are deemed free.

Transfections derive their name from the corresponding biochemical technique of inserting genetic material into cells. Transfections are similar to substitutions in that they replace one fragment with another, with the difference being that the replacement fragments are sourced from the molecule population instead of the fragments database. Hence, they exploit the internal variability of the population, fulfilling a similar role to crossover operators in traditional genetic algorithms. We opted out of traditional crossover operators because crossing over graphs is non-trivial. Historically graph crossover operations have been implemented as exchanges of subgraphs [10, 35, 37, 38], or of side chains around a maximum common substructure [30, 47]. If we were to implement one of these approaches it would have to operate on our chromosomal meta-graph without infringing on the connection compatibility rules. The former approach would be error prone, whereas the latter assumes the presence of a large common substructure, which is unlikely if the fragments are diverse and the number of fragments is large. While the transfection operator is less disruptive than a crossover operator, the unidirectional flow of genetic material in transfections is easier to implement, guarantees the success of the operation and reduces the time complexity from O(n^2^) to O(n) compared to a bidirectional crossover.

Translations/rotations move a fragment from one position and orientation to another within the same molecule. They operate similar to a deletion and expansion in tandem. By inserting the fragment back in its starting position but with a different orientation it can effectively be rotated in place.

Lastly, for those scoring functions operating on 3D molecular structures, a stereochemistry flip operator is available. This operator chooses a random chiral atom or stereochemical double bond and inverts its stereochemistry.

#### Connection rules enforcement

**Fig. 5:**
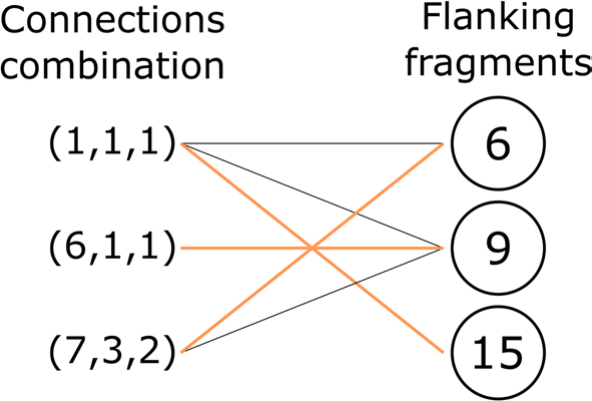
MBPM constructed to query whether a hypothetical fragment with a given set of connectors (left) is compatible with a combination of fragments (right). Black and orange edges represent compatibility relationships. The solution to the MBPM (i.e. the matching) is shown as the orange highlighted edges. Since the cardinality of the matching is equal to the number of flanking fragments our hypothetical fragment is compatible

LEADD’s genetic operators satisfy the connection compatibility rules by searching for fragments that can bond simultaneously to a given combination of fragments. Whether a specific query fragment fulfils the above condition can be expressed as a Maximum Bipartite Matching problem (MBPM). We construct the bipartite graph by placing the query fragment’s free connectors in one vertex set, and the fragments within the combination in the other vertex set (Fig. 5). The edges between both vertex sets are drawn according to the lax connection compatibility rules (Fig. 2B), with an edge representing that a connection is compatible with a fragment. This MBPM is then solved with a modified version of the Hopcroft-Karp algorithm [48]. The standard version of the algorithm is deterministic and always returns the same matching, even if multiple matchings with the same cardinality exist. By randomizing the order in which it iterates over vertices and edges it returns a random maximum cardinality matching instead. If the cardinality of the resulting matching is equal to the number of fragments within the combination, the query fragment is compatible with said combination of fragments.

To find all fragments that could bond to a combination of fragments one must interrogate all candidate fragments separately, which entails solving MBPM multiple times. This is computationally reasonable when the number of candidates is small, namely during internal deletions, transfections and translations/rotations. However, it becomes unreasonable for operations that sample fragments from the large fragments database, namely expansions and substitutions.

**Fig. 6:**
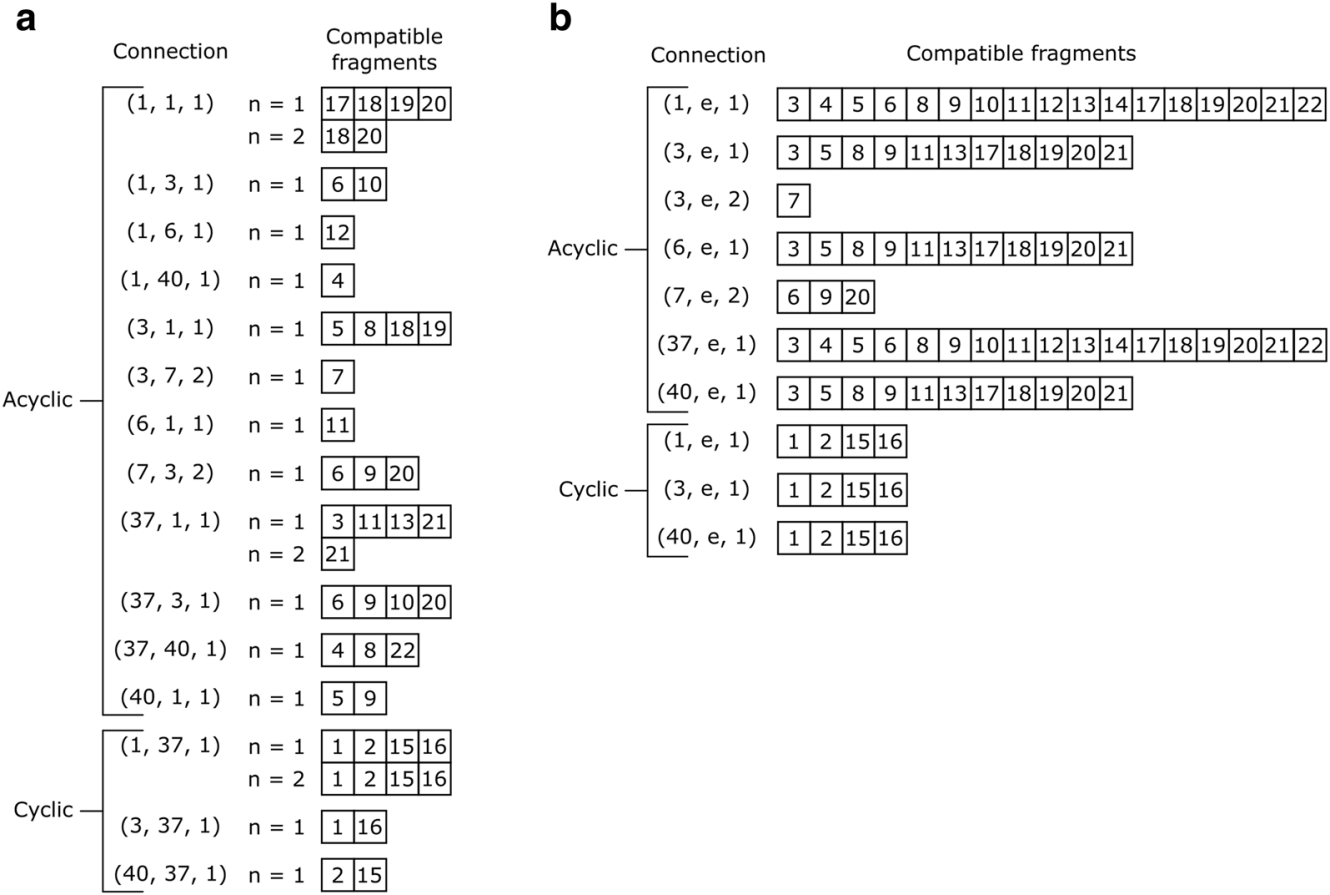
Connection-fragment compatibilities of the fragments in Fig. 1 according to (**a**) the strict compatibility rules and (**b**) lax compatibility rules, as described in Fig. 2. Fragment weights are omitted for clarity purposes (Additional file 1: Fig. S2). Fragments are stratified according to their cyclicity, and in the case of the strict compatibility definition (**a**) also according to how many instances (n) of the connection the fragment has. In (**b**), “e” denotes any ending atom type. Note that in (**a**) higher strata are subsets of the lower strata, and that (**a**) is a subset of (**b**)

In those cases, we solve the problem through Multiple Set Intersection (MSI). Before LEADD is executed we precompute which fragments are compatible with each connection according to the strict connection compatibility rules and store their IDs in sets (Fig. 6A). Since a connection combination may have repeats of the same connection, the compatible fragment IDs are stored stratified according to how many instances of compatible connections they have. If a fragment is compatible with *n* instances of a connection it is also compatible with *1* to *n−1* instances. To be able to control the number of ring fragments within the designed molecules, fragments are also stratified according to whether these are cyclic or acyclic.

**Fig. 7:**
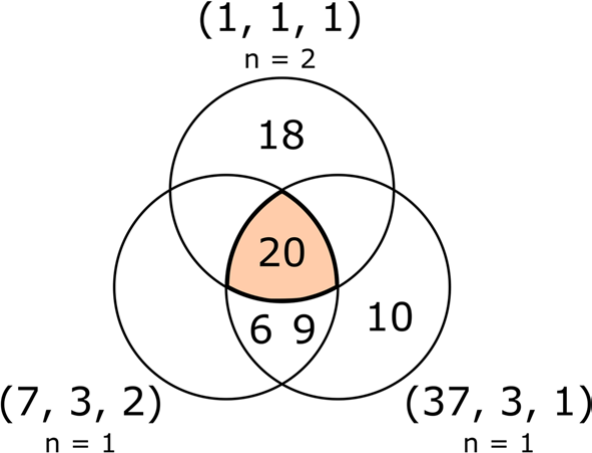
Venn diagram of the multiple intersection result for acyclic fragments compatible with the connections combination [(1,1,1), (1,1,1), (7,3,2), (37,3,1)], using the precalculated compatible fragments according to the strict compatibility definition (Fig. 6a)

At runtime these arrays are loaded, and the list of fragments compatible with a combination of connections is calculated as the intersection of the fragment IDs compatible with each of its connections separately (Fig. 7). Note that since fragments may have more than one free connector, if we wish to find fragments compatible with a combination of fragments, we must define all unique combinations of their free connectors and solve the MSI problem for each of them. The final result is the union of all resulting sets.

The MSI connection-fragment compatibilities must be computed using the strict connection compatibility definition to ensure that the same connector doesn’t contribute to a fragment showing up in more than one set of compatible fragments. Because of this, the MSI approach returns a subset of all fragments that would be deemed compatible according to the MBPM approach (Fig. 6). Nonetheless, the final orientation of fragments retrieved with the MSI approach can still be determined through MBPM.

#### Operation outcome sampling

In the event that an operator finds multiple suitable operation outcomes a random one is chosen, typically through roulette wheel selection. For expansions, deletions and substitutions the weight *W* of a fragment *F* is calculated based on its frequency *q* in the database and its size *N*, in numbers of heavy atoms, according to the following equation:

1 $$W_{F} = q_{F}^{\gamma } \cdot N_{F}^{\lambda }$$

where the exponents *γ* and *λ* are user parameters. *γ* determines how much the fragment selection should be guided by the fragment frequencies, with the default being *γ* = *1*. If the user wishes true random fragment selection this can be done by setting *γ* = *0*. *λ* is a size biasing term intended to be used when mixing fragments of different sizes. For efficiency reasons they are precalculated and stored alongside the connection-fragment compatibilities (Additional file 1: Fig. S2).

For transfections the weight is calculated following the same formula but with the score *S* of the fragment’s owner molecule *R* as an additional variable term:

2 $$W_{F} = q_{F}^{\gamma } \cdot N_{F}^{\lambda } \cdot S_{R}^{\zeta }$$

where *ζ* is a user specified parameter signifying the transfection bias towards fragments contained in high scoring molecules.

The translation/rotation and stereo flip operators select operation outcomes through uniform random sampling instead.

#### Cyclicity control

Fragment identity comprises both the molecular graph and connectors. Generally, the number of unique fragments increases with (1) the size of the fragments and (2) the atom type and connector diversity (Additional file 1: Table S1). Differences in fragmentation procedure between acyclic and cyclic regions of source molecules can cause imbalances in the number of unique fragments, as well as their frequencies, which can lead to fragment sampling biases. Since cyclic fragments tend to outnumber their acyclic counterparts (Additional file 1: Table S1), if fragments were sampled uniformly (γ = 0, Eq. 1) it would be more likely to sample cyclic fragments. Conversely, under weighted sampling (γ > 1), and when defining acyclic fragments as subgraphs of *s* > *0,* certain acyclic atoms are represented in more than one fragment. Since ring systems aren’t fragmented, this causes an overrepresentation of acyclic atoms in the fragment frequencies with respect to the cyclic ones. If these factors aren’t accounted for during fragment sampling, we risk designing either very rigid or very flexible and non-druglike molecules.

To circumvent this issue the genetic operators with the capacity to modulate the number of ring atoms in a molecule (*N*_*r*_), namely expansions, deletions, substitutions and transfections, decide whether and how *N*_*r*_ ought to be changed prior to selecting a suitable acyclic or cyclic fragment to do so, according to the current *N*_*r*_.

How the operator will modulate *N*_*r*_ is based on the probabilities returned by up to two functions operating in tandem. In first instance, a Gaussian function describes the probabilities of keeping *N*_*r*_ constant. For expansions and deletions this function suffices to decide how to modulate *N*_*r*_. However, for substitutions and transfections, if in the preceding step it was decided to change *N*_*r*_, a second logistic function returns the probability of increasing *N*_*r*_. Further details can be found in the supplementary material.

### Lamarckian evolution guidance

Given that the database fragment weights are static, so are the likelihoods of genetic operation outcomes, regardless of whether the same or similar operations proved beneficial or not in the past. In an attempt to improve the efficiency of the algorithm, as an extension, we conferred it with a certain ability to “learn” from the outcomes of previous genetic operations in hopes of increasing the likelihood of carrying out productive operations in the future. To this end, each connector within a molecule is endowed with a pair of arrays: one storing the IDs of compatible fragments *F* and one storing their corresponding weights *W*_*F*_. The weights array is initialized to a copy of the database fragment weights (Additional file 1: Fig. S2), but it’s free to change with each generation.

During evolution, a copy of a parent molecule *P* is subjected to a genetic operation, targeting some fragment *V*, to generate a child molecule *C*. The score *S* of *C* is compared to that of *P*:

3 $$\Delta S = S_{c} - S_{P}$$

Molecules keep track of which fragments were placed and/or removed from each connector during the operation. For each connector involved in the operation, based on the nature of the operation and its outcome (Table 1), the weights array of both the *P* and *C*’s connectors are modified according to the following expression:

4 $$W_{F} = W_{F} \cdot \left( {1 + g \cdot l \cdot Tc_{FV} } \right)$$

**Table 1:** Learning rate sign of Eq. 4 for bond creations (i.e. attaching a fragment to a connector) and destructions (i.e. deleting a fragment from a connector) based on the score change associated with the operation

Operation	ΔS	Learning rate sign (*g*)
Bond creation	> 0	+ 1
	≤ 0	− 1
Bond destruction	> 0	− 1
	≤ 0	+ 1

where *g* is the reinforcement sign, *l* is a user-specified reinforcement rate and *Tc*_*FV*_ is the Tanimoto topological similarity coefficient of fragments *F* and *V* according to ECFP4 fingerprints [42]. For performance reasons, all pairwise fragment similarity coefficients are precalculated and stored as a square symmetrical matrix in a HDF5 file [49].

Whether the change in weight is positive or negative (*g*) depends on the nature of the operator and the change in score (Table 1). LEADD maximizes strictly positive scores. The general principle is that if a newly placed fragment at a given connector increased the molecule’s score (i.e. improved the score), the weights of similar fragments are increased, whereas if it stayed the same or decreased, the weights of similar fragments are decreased. The opposite paradigm is true for fragments being removed from a given connector.

This guided evolution serves two purposes. On one hand it can accelerate convergence by focusing the sampling on fragments that have been shown to be associated with good scores. On the other hand, since weights of similar fragments are decreased also when the score doesn’t change, given enough time it could help the algorithm in escaping local fitness minima.

One could interpret a molecule’s connectors’ weights arrays as its reproductive behavior or its memory regarding which chemotypes at which positions are linked to better scores. Parents adapt their reproductive behavior to increase the likelihood of generating fit offspring based on the outcome of their previous reproductive events. Hence, the reproductive behavior is an acquired trait. This, coupled to the fact that the connector arrays are an integral part of the chromosome, and therefore inherited by the offspring, constitutes a Lamarckian evolutionary mechanism.

### Evolutionary algorithm

Over the course of a number of generations (or until some convergence criterion is met) the molecules within the population are bred to generate offspring. Each generation a number of parent molecules is chosen to generate an equal number of child molecules. Parents reproduce asexually, and the same parent may reproduce more than once in the same generation. A copy of the chosen parent is subjected to a genetic operator to yield the child molecule. Molecules are chosen to be parents through fitness proportionate selection, with the weight of a molecule *R* being given by Eq. 5. Note that the *ζ* parameter takes the same value as in Eq. 2.

5 $$W_{R} = S_{R}^{\zeta }$$

Optionally, the user may enforce population topological diversity through means of an internal similarity filter. The topological similarity between two molecules is calculated as the Tanimoto coefficient between their ECFP4 fingerprint [42]. If the similarity of a child molecule to any of the current members of the population surpasses a given threshold, the child is discarded. Otherwise, it’s added to the population.

The child molecules are scored, and a specified number of best scoring molecules within the population, including parents, is retained. If guided evolution is enabled the connector weights are adjusted based on the change in score caused by the operation. Lastly, the surviving molecules are fed to the next generation of the algorithm.

While the use of fragments and connection compatibility rules is meant to reduce the likelihood of designing synthetically unfeasible molecules, this may not be sufficient to achieve this goal. For users wishing to consider synthetic accessibility on a higher level a SAScore [18] filter and heuristic score modifier [14] are provided.

A flowchart of the algorithm can be found in Additional file 1: Fig. S4.

### API

LEADD is scoring function agnostic, the only requirements being strictly positive floating point molecule scores, with higher scores being better. The recommended way of coupling a problem-specific scoring function is using the C++ or Python API. An instance of a molecule design class is initialized using a settings file and output directory path. This class has member functions to expand the population with children, get their SMILES, set their scores, and wrap up the generation by selecting the fittest individuals. A Python example of how these functions can be used in conjunction with a user scoring function *ScoreMolecule()* is shown below.
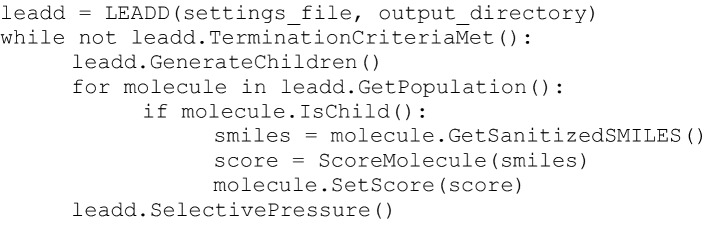


### Benchmark

LEADD’s performance was evaluated with the goal-directed GuacaMol benchmark suites [13]. Specifically, we used the “trivial” and “version 2” (V2) benchmark suites. Briefly, these benchmark suites consist of 7 and 20 objective functions respectively that assign scores between 0 and 1 to populations of molecules. The overall score of the benchmark suite can be calculated as the sum of all individual benchmark scores. We chose to include the trivial benchmarks in our analysis because the majority of the V2 objective functions point towards topologies of known and synthetically feasible drugs. Hence, the objective functions implicitly provide some notions of drug-likeness, potentially occluding some SA issues.

For standardization purposes we used GuacaMol’s training set, which is a subset of ChEMBL [50], as fragmentation input. Fragment databases were created for each investigated combination of fragmentation and atom typing scheme (Additional file 1: Table S1).

The benchmark suite was used to find a set of reasonable default parameters for LEADD. Given the large number of parameters an exhaustive parameter exploration was unfeasible. We resorted largely to a trial-and-error approach. Some parameters, including the population size and convergence criteria were fixed. Additionally, since LEADD requires a guess of the number of ring atoms in the ideal solution, where possible, we used the benchmark goals to set reasonable values for these parameters (Additional file 1: Table S2). The rest of the parameters were sorted according to their perceived importance. For parameters assumed to be uncorrelated we tested multiple values for each one and fixed it to the value that yielded the best results. If this wasn’t the case, we evaluated combinations of the correlated parameters in a multi-factorial design.

Ten replicas were ran for each combination of settings. Benchmark scores and SAScores of designed molecules were taken as OP and SA metrics respectively. ChEMBL [50] feature counts were used for SAScore calculations. For statistical analysis the results of all benchmarks were pooled per setting. Since OP was found to be distributed non-normally, differences in it were evaluated with non-parametric statistical tests: either the Wilcoxon-Mann–Whitney U-test [51] or the Kruskal–Wallis [52]/Schreirer-Ray-Hare [53] H-test followed by pairwise Conover-Iman tests [54] with Šidák correction [55]. SAScores were distributed normally and analyzed with t-tests or one- or two-way analysis of variance (ANOVA) with interaction followed by Tukey’s Honestly Significant Differences test. α = 0.05 was taken as significance level and family-wise error rate (FWER) for all tests.

LEADD’s performance was compared to that of GB-GA [35], an atom- and graph-based genetic algorithm for molecular design which has previously been shown to be a powerful optimizer [13, 23], and a standard virtual screen of GuacaMol’s training set using the benchmark’s objective function. GB-GA’s mutation rate was set to the default 0.01. Both algorithms used a population size of 100 and were granted a maximum of 10,000 generations. Evolution terminated prematurely after a number of generations without improvements in the population’s scores: 1000 for LEADD and 5 for GB-GA. We explored granting GB-GA 1000 generations without improvement but found that its lack of convergence guards caused the population diversity, and ultimately the benchmark scores, to degrade during long runs.

## Results and discussion

LEADD was found to be quite robust to changes in most of its construction parameters, as different values didn’t influence its performance greatly. As an exception, LEADD was sensitive to the internal similarity threshold since it’s the algorithm’s main premature convergence guard (data not shown). LEADDs base parameters can be found in Additional file 1: Table S3. Fragmentation parameters had larger effects on both OP and SA of designed molecules.

### Effect of atom typing scheme

**Fig. 8:**
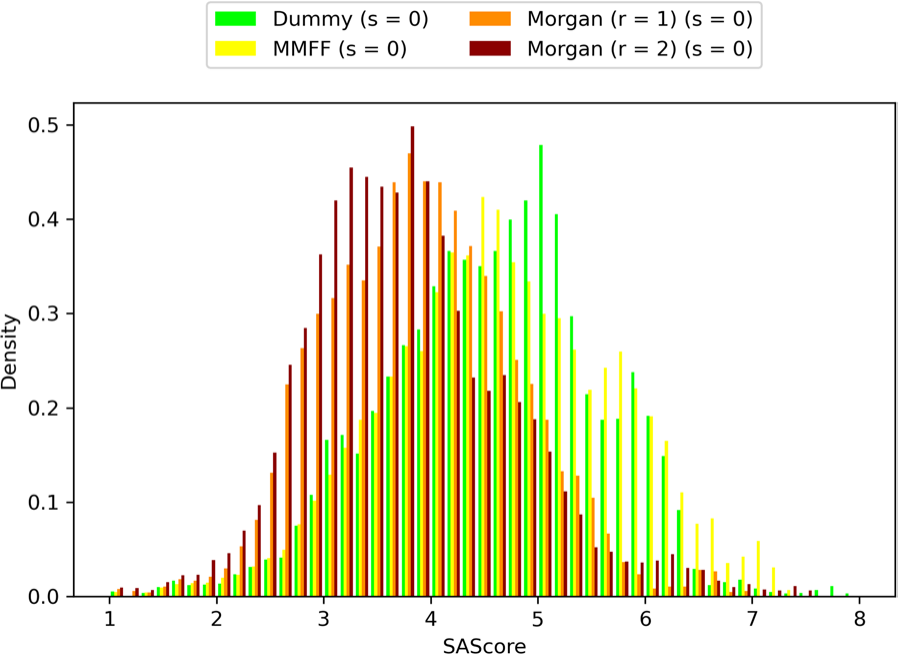
Comparison of designed molecules’ SAScore distributions using different atom typing schemes. Includes molecules of all benchmarks and replicas. Molecules with lower SAScores are predicted to be easier to synthesize

One of the main questions we wanted to answer was if the knowledge-based atom compatibility rules aided the algorithm in designing SA molecules. To that end, we measured the SAScores of molecules designed using the MMFF and Morgan (r = 1 and r = 2) atom typing schemes. As a control, we included “dummy” atom types (i.e. all atoms have the same atom type), whereby all connections with the same bond order are compatible. All tests used single-atom acyclic fragments (s = 0). Molecules with lower SAScores are predicted to be easier to synthesize. Figure 8 shows that molecules designed with Morgan atom types, regardless of the radius, have lower SAScores than those designed with dummy or MMFF atom types. Differences between all other pairs of atom typing schemes were of little practical significance (Additional file 1: Table S4). It’s interesting to note that the mean SAScore values for Morgan atom types fall well below 4.5, which has been suggested as a cut-off for easy to synthesize molecules [19]. By contrast, the mean SAScore values for dummy and MMFF atom types are approximately 4.6.

**Fig. 9:**
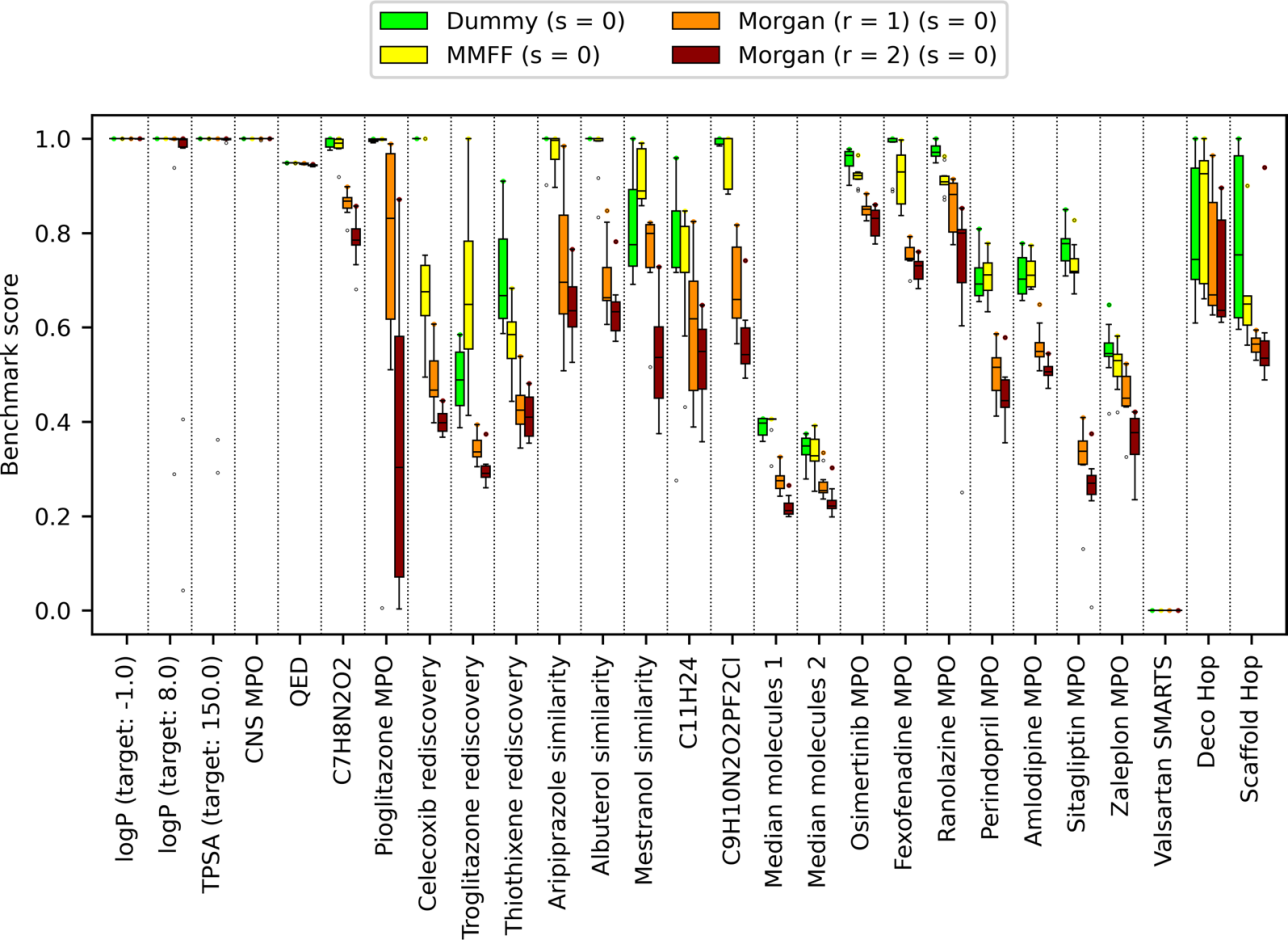
LEADD optimization power comparison between atom typing schemes. Benchmark scores range between 0 and 1, with higher scores being better. Boxes represent interquartile ranges (IQR), the black line within them medians and the whiskers Q ± 1.5IQR. Data beyond the whiskers are considered outliers and represented as dots. Colored dots represent maximum benchmark scores

Unfortunately, we also noted that Morgan atom types were associated with significantly lower OP compared to dummy and MMFF atom types (Fig. 9). The differences between dummy and MMFF atom types and between Morgan atom types of different radii were not statistically significant (Additional file 1: Table S5).

Taken together these results suggest that the choice of atom typing scheme defines a trade-off between OP and SA. The chemical diversity of atomic environments is vast, and classifying them into a small number of atom types means that atom typing schemes are degenerate, much like the human genetic code. The number of distinct atom types can be taken as an approximate measure of the scheme’s degree of degeneracy. LEADD tries to replicate the molecular connectivity of molecules seen in a library of drug-like molecules, but if a very degenerate atom typing scheme mischaracterizes this connectivity the algorithm’s ability to replicate it falters. In our fragment databases we recorded 64 MMFF, 14,811 Morgan (r = 1) and 381,252 Morgan (r = 2) atom types (Additional file 1: Table S1). Unique Morgan atom types greatly outnumber their MMFF counterparts, explaining the better SA associated with them.

The atom typing scheme’s degree of degeneracy also defines the observed OP-SA trade-off. LEADD considers two atom types to be compatible, and therefore suitable for bonding, if they have been observed bonded in reference molecules at least once. Given the same set of reference molecules, the probability of observing any specific pair of atom types bonded is larger when the number of distinct atom types is small. Consequently, the more degenerate an atom typing scheme, the more promiscuous its atom types, in the sense that atom types will be deemed compatible with a larger number of other atom types. Ultimately, this also affects the number of fragments that are compatible with each connection. In the case of MMFF atom types, 85.96% of all fragments are compatible with the average connection according to the lax compatibility definition. This number drops to 1.53% and 0.05% for Morgan (r = 1) and Morgan (r = 2) atom types respectively. Even more dramatic differences are observed when considering the strict compatibility definition (Additional file 1: Table S1). This highlights that atom type promiscuity enables the algorithm to access a larger number of states (i.e. molecules) from the current state, aiding it in the escape of local fitness minima and explaining the associated greater OP.

Out of the tested atom typing schemes, we believe that for most use cases Morgan (r = 1) atom types represent the best OP-SA compromise. Other compromises of interest may be achievable with alternative atom typing schemes. LEADD can be readily expanded to use other atom typing schemes. For instance, one could collapse Morgan atom types into a smaller number of atom types with some type of hashing function. However, as this would inevitably cause collisions, the hashing function would need to be locality sensitive to avoid merging completely unrelated atom types. An alternative approach might be to cluster atomic environments and use cluster assignments as atom types. This approach could allow fine control over the OP-SA trade-off by modulating the number of clusters. We would like to remark however that the number of unique atom types is only a good metric for atom typing degeneracy when atomic environments are distributed uniformly across atom types. This is likely to be the case for Morgan atom types since they are calculated using hashing functions, which are designed to distribute inputs uniformly over an integer range, but may not be the case for other schemes. Instead, it would be more appropriate to use metrics that measure the information content of atom types (i.e. within atom type atomic environment similarities).

### Implications of compatibility binarization

LEADD’s approach to find suitable fragments for genetic operators requires that connection compatibility be expressed as a binary property. However, it may be argued that connection pairs are on a compatibility spectrum based on the observed frequency of said pair: if a pairing is observed thousands of times it’s more compatible than if it’s observed just once, yet they are deemed equally compatible. Consequently, infrequent connections may misrepresent molecular connectivity. We regularly observed large disparities among compatible connection pairing frequencies and wanted to measure the extent to which this is detrimental to the SA of designed molecules. By default the MBPM approach uses the lax compatibility definition, but this may be changed to the strict definition. Under the strict compatibility definition each connection is compatible with exactly one other connection, eliminating compatible connection pairing frequency imbalances. We found no practically significant differences in SAScore when using the strict compatibility definition for MBPM as opposed to the lax one (Additional file 1: Fig. S5). Considering that a fragment’s connectivity is part of its identity, infrequent connections are contained to infrequent fragments. Since LEADD samples fragments with a probability proportional to their frequency we hypothesize that, while the binarization of connection compatibility does misrepresent the molecular connectivity of the reference library, this rarely manifests itself in designed molecules.

### Effect of fragmentation scheme

**Fig. 10:**
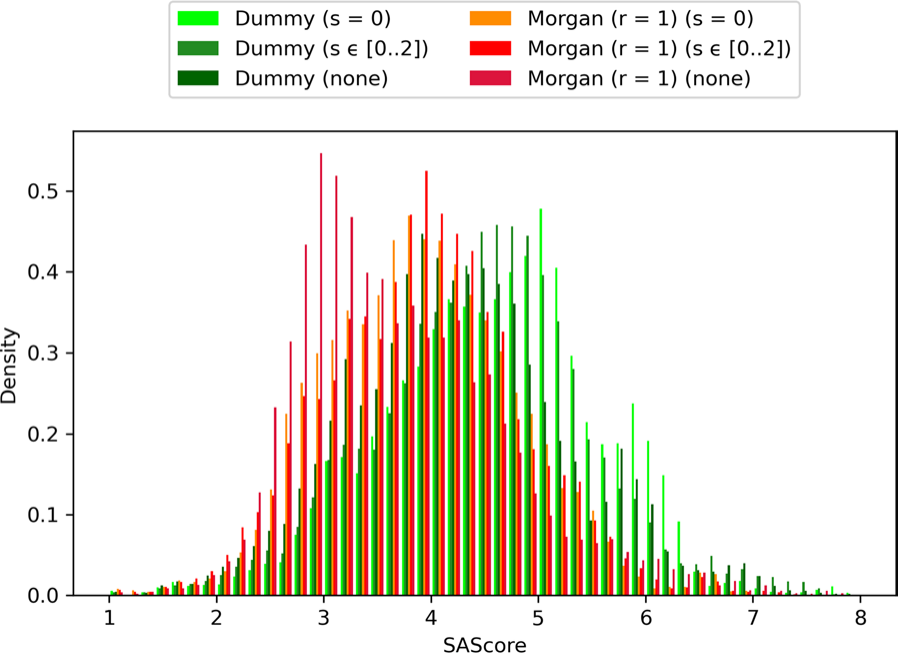
Comparison of designed molecules’ SAScore distributions using different atom typing schemes. Includes molecules of all benchmarks and replicas. Molecules with lower SAScores are predicted to be easier to synthesize

The atom typing scheme degeneracy, the binarization of connection compatibility, and other factors such as connection compatibility being expressed only as pairwise relationships, all contribute towards LEADD’s description of molecular connectivity being imperfect. Each bond created by the algorithm has a probability of being non-drug-like. While we have discussed approaches to decrease this probability, an alternative approach to improve the drug-likeness of designed molecules is to reduce the number of bonds created by the algorithm. This can be achieved using larger fragments. To prove this we ran the benchmark using different types of acyclic fragments: single-atom fragments (s = 0), fragments with 0 to 2 bonds (s ϵ [0 .. 2]) and whole side chains and linkers resulting from the deletion of ring systems. In general, the SAScores of molecules designed using larger fragments were lower than those designed using smaller fragments (Fig. 10). While the SAScore differences between s = 0 and s ϵ [0 .. 2] were almost negligible, using monolithic acyclic fragments did lead to substantial improvements in SAScore (Additional file 1: Tables S6, S7). It’s interesting to note that the observed improvements in SAScore were larger for dummy atom types than for Morgan atom types, highlighting that the bonds created when using Morgan atom types are more drug-like.

**Fig. 11:**
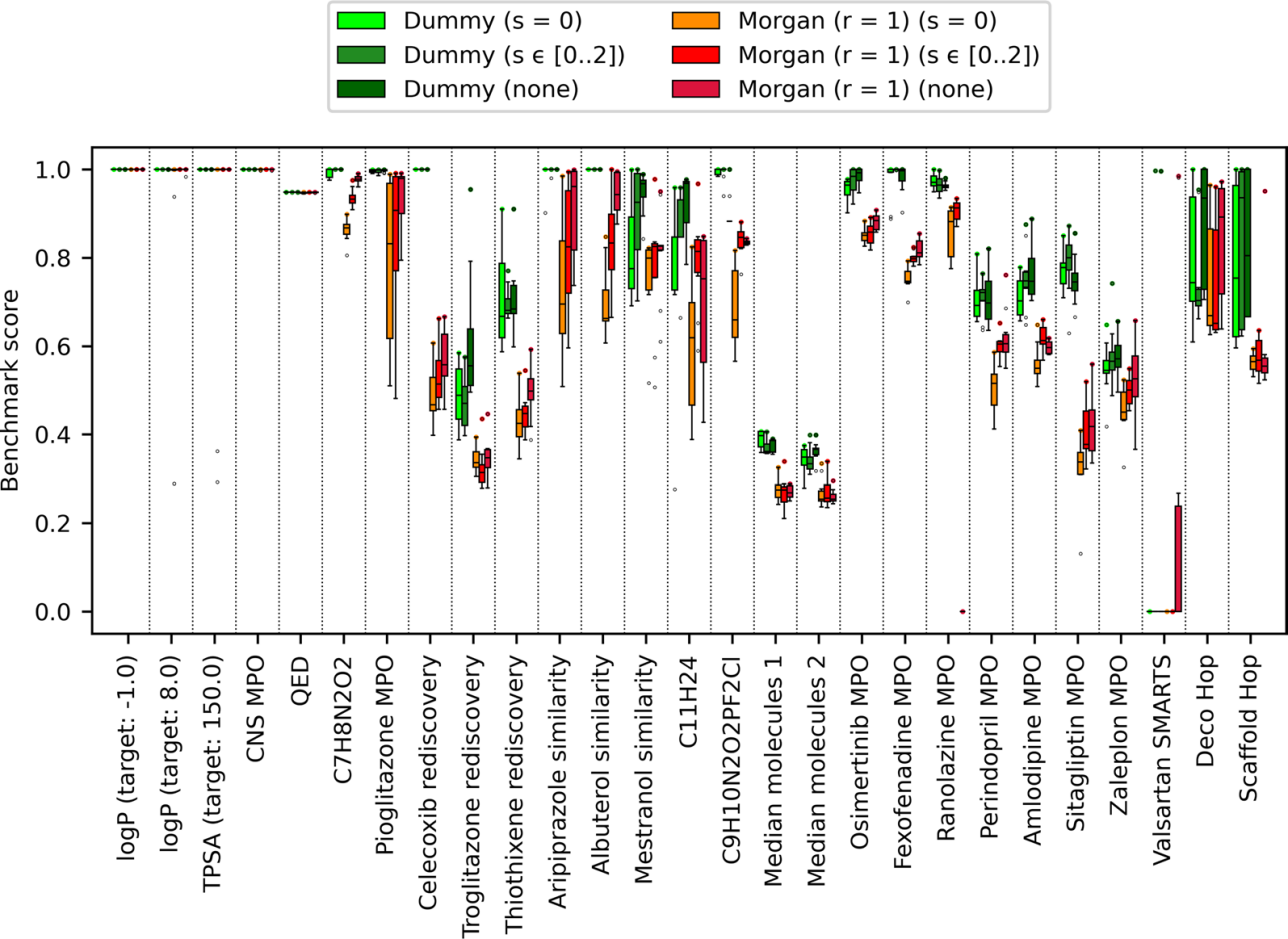
LEADD optimization power comparison between different combinations of atom typing and fragmentation schemes. Benchmark scores range between 0 and 1, with higher scores being better. Boxes represent interquartile ranges (IQR), the black line within them medians and the whiskers Q ± 1.5IQR. Data beyond the whiskers are considered outliers and represented as dots. Colored dots represent maximum benchmark scores

The use of larger fragments didn’t affect LEADD’s OP when using dummy atom types. However, we did observe significant improvements in OP when using large fragments coupled with Morgan (r = 1) atom types (Fig. 11, Additional file 1: Tables S8, S9). Genetic operations using larger fragments are associated with bigger step sizes in chemical space, which allows the algorithm to escape local fitness minima. Because the number of chemical states accessible from a given state is much smaller when using Morgan atom types as compared to dummy atom types, the probability of getting stuck in local fitness minima is larger in the former case. This explains why a bigger step size is beneficial for Morgan, but not dummy atom types. It’s worth noting that the step size associated with larger fragments isn’t longer solely because of the bigger number of atoms per fragment, but also due to the greater degree of branching in larger fragments. While we implemented internal operators that attempt to mitigate this, there still is a risk that the algorithm may design certain highly branched topologies that are difficult to modify with genetic operators without unwinding the entire stack of operations. Since large fragments can capture branched motifs as a single unit, the risk of this happening is reduced. Future algorithms could improve upon this by implementing operators that target entire sections or branches of the meta-graph instead of a single vertex.

Given that larger fragments improve SA and either increase OP or don’t affect it, it’s tempting to conclude that the use of large fragments is always preferable. However, it should be noted that the larger step sizes associated with big fragments also carry the risk of “jumping” over good solutions. This can be partially overcome by mixing fragments of different sizes (e.g. s ϵ [0.. 2]). A more pressing issue is that the use of large fragments requires a very extensive and diverse library of fragments to adequately represent chemical space. Besides dictating greater amounts of memory to store the pre-calculated compatible fragments, as the number of fragments grows so does the size of the search space, and with it the number of operations and generations necessary to adequately explore it. For Morgan atom types, we believe that the improved SA and OP tied to monolithic fragments justify their use. However, for dummy atom types we think that the minor SA improvements aren’t sufficient justification.

### Handling fragment numerosity

A large number of fragments also poses the question of how to prioritize fragments to explore chemical space efficiently. We opted to use the fragments’ frequencies in drug-like matter as biasing weights to determine the outcomes of genetic operations. In an attempt to improve upon this, we also implemented a Lamarckian evolutionary mechanism that biases the exploration towards certain areas of the search space based on the outcomes of previous operations. A similar concept was explored in the particle swarm optimizer Colibree [56], where each molecule has preferences towards certain fragments, encoded as a floating point number array. In Colibree these preferences apply to the entire molecule, which is computationally more efficient and enables straightforward communication of preferences among molecules within the swarm, but lacks the spatial resolution that one would desire when working with structure-based scoring functions. Our Lamarckian evolutionary mechanism attempts to improve on this by assigning fragment preferences to connectors instead. Unfortunately, with the explored settings, the Lamarckian guided evolution mechanism failed to significantly improve the optimization power of the algorithm (data not shown). One possible explanation for the shortcomings of the approach is that, given the large number of fragments (10^5^–10^6^ compared to the 7196 of Colibree), the number of generations for which LEADD runs (i.e. 1000–10,000) is insufficient to resolve connector-fragment preferences. The impermanence of connectors may exacerbate the problem. When a fragment is deleted or substituted the knowledge accumulated in its connector arrays is erased, effectively resetting the progress of the Lamarckian evolution. A potential solution could be mapping fragment preferences to points in space instead, which also would allow molecules to share their preferences among each other. However, the observed slower runtimes and larger memory footprints discourage us from exploring this approach further.

### Comparison of SA improvement approaches

**Fig. 12:**
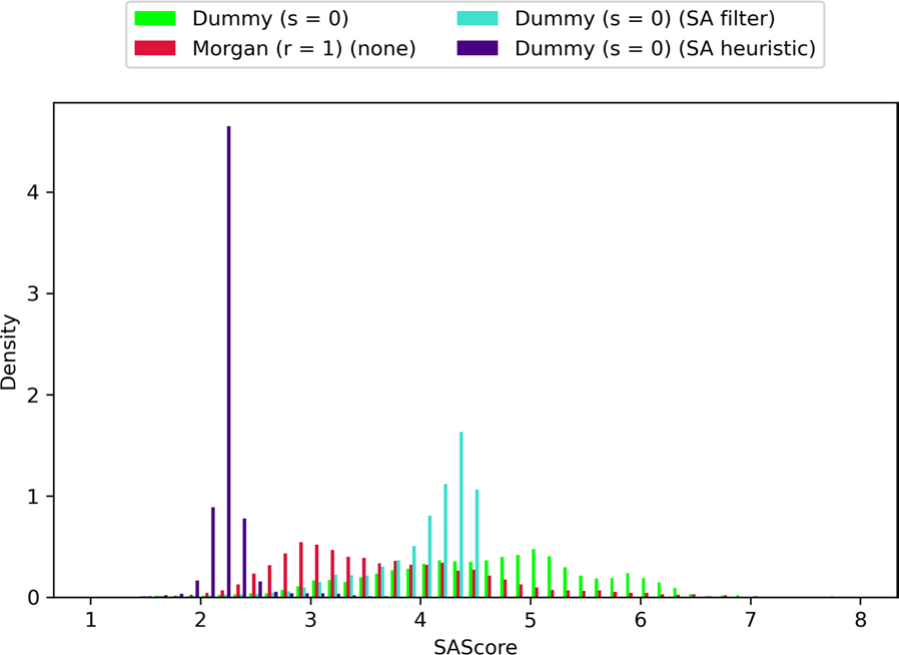
Comparison of designed molecules’ SAScore distributions using different SA optimization strategies. Includes molecules of all benchmarks and replicas. Molecules with lower SAScores are predicted to be easier to synthesize

**Fig. 13:**
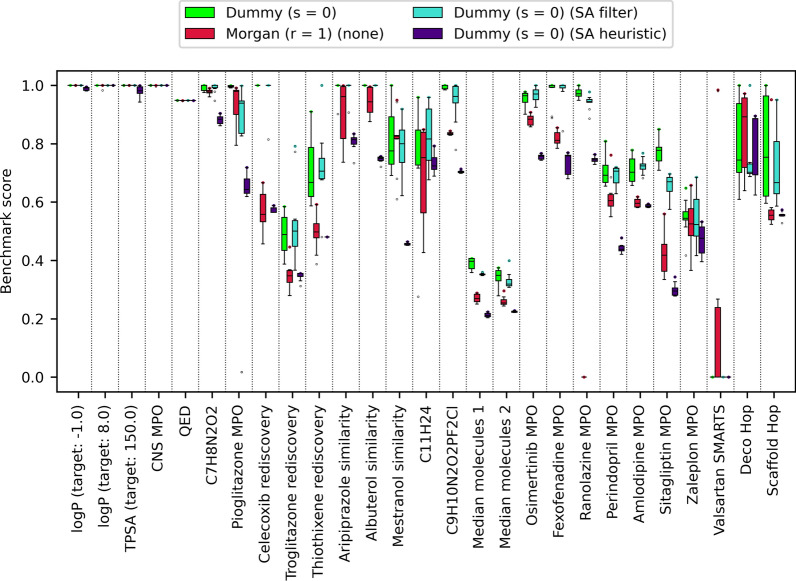
LEADD optimization power comparison using different SA optimization strategies. Benchmark scores range between 0 and 1, with higher scores being better. Boxes represent interquartile ranges (IQR), the black line within them medians and the whiskers Q ± 1.5IQR. Data beyond the whiskers are considered outliers and represented as dots. Colored dots represent maximum benchmark scores

LEADD also ships with more traditional means of improving the SA of designed molecules, namely a simple filter that deletes molecules with SAScores above a given threshold and a SAScore-based heuristic score modifier that biases the objective function towards molecules with lower SAScores, as described by Gao and Coley [14]. As a reminder, the SAScore is a composite metric based on (a) how much the molecular connectivity of a molecule resembles that of reference drug-like molecules (i.e. FeatureScore) and (b) the number of synthetic nuisances within that molecule, for example stereo centers, spiro-, bridged- and macro-cycles (i.e. ComplexityPenalty). Because the atom type approach to increase SA only tries to improve the FeatureScore it can be of interest to combine it with the SAScore filter or heuristic. We were interested in comparing how these different approaches to increase SA fare on their own. The parameters for the SAScore filter (SAScore ≤ 4.5) and heuristic (µ = 2.23, σ = 0.65) were taken from the literature, where they have been described as effective means to design SA molecules [14, 19]. Our results confirm that all approaches can be used to design more SA molecules (Fig. 12, Additional file 1: Table S10) and that, with the exception of the SAScore filter, this was accompanied by a significant loss of optimization power (Fig. 13, Additional file 1: Table S11). There appears to be an inverse correlation between SA and OP, and the observed OP-SA compromises seem to define a FeatureScore Pareto front (Additional file 1: Fig. S6). However, it should be noted that each approach has a different SA target. We didn’t manage to find SAScore filter and heuristic parameters that replicate the SAScore distribution of Morgan (r = 1) atom types. Hence, which approach provides the best OP-SA trade-off, if any, is inconclusive.

### Comparison to other algorithms and virtual screening

**Fig. 14:**
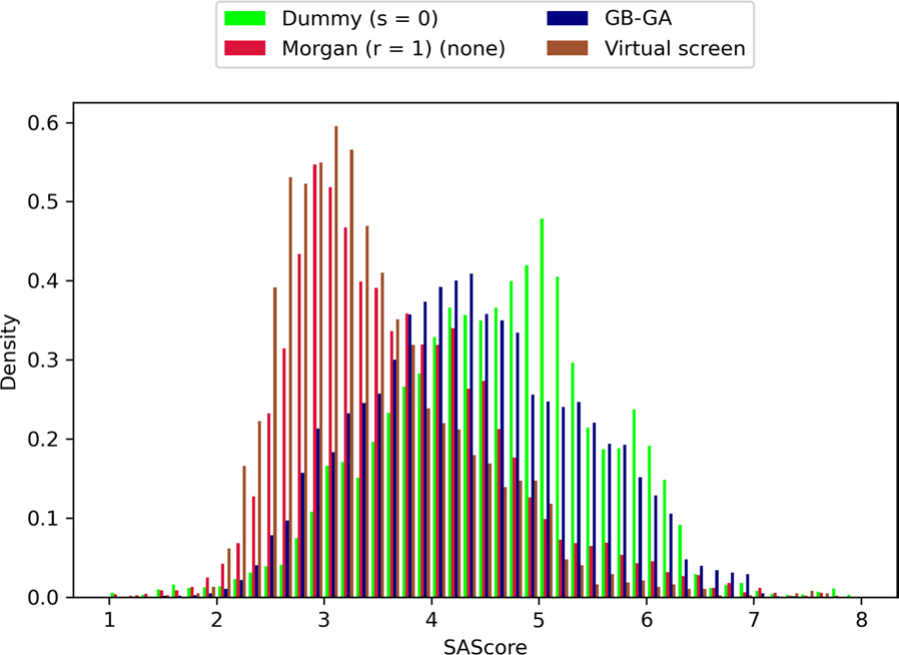
Comparison of SAScore distributions between molecules designed by LEADD and GB-GA and those found through a VS. Includes molecules of all benchmarks and replicas. Molecules with lower SAScores are predicted to be easier to synthesize

Lastly, we wanted to compare LEADD’s performance to that of GB-GA [35] and a VS of the GuacaMol virtual library. In terms of OP, LEADD with dummy atom types outperformed the VS in 26/27 benchmarks, with the only exception being the Valsartan SMARTS benchmark which uses a binary scoring function ill-suited for goal-directed optimization approaches. LEADD with the use of dummy atom types is comparable to GB-GA, in the sense that both are graph-based EAs with very few restrictions on how atoms can be connected. Correspondingly, the SA (Fig. 14, Additional file 1: Table S12) and OP (Fig. 16, Additional file 1: Table S13) of these two systems are comparable. The key difference between both algorithms is that LEADD modifies molecules on a fragment level as opposed to the atom level of GB-GA. Although we paired dummy atom types with single-atom acyclic fragments, ring systems are always treated as monolithic fragments. We expected this to yield improved SA and smaller OP, yet found the opposite. LEADD has better OP than GB-GA, outperforming it in 18/27 benchmarks and performing equally well or better in 23/27 benchmarks. We attribute this to the bigger step size associated with fragments and the internal topological similarity threshold to enforce population diversity, giving it an edge at escaping local fitness minima. It’s also possible that the same factors explain the better SA of molecules designed by GB-GA. Most GuacaMol benchmarks incorporate topological similarity to a reference drug-like molecule in their objective functions, implicitly capturing some SA notions. Because of LEADD’s internal similarity threshold only the best individual within the population can assume the identity of the reference molecule, whereas the rest are forced to diverge from it. In GB-GA all individuals are allowed to approach the target molecule as much as possible, benefitting to a greater extent from the implicit SA target of the objective function. Moreover, GB-GA doesn’t allow the creation of SSSR cycles bigger than six-membered rings whereas some of the fragments used by LEADD do include bigger cycles. Since the SAScore incorporates a macrocycle penalty this could account for some of the observed differences. Ultimately, the magnitude of the SA changes associated with the use of fragments, be it cyclic or acyclic, are small (Additional file 1: Tables S7, S12). This calls into the question the widespread practice of fragment-based molecular construction as a means to improve SA, and we hypothesize that its effectiveness depends on how well in silico fragments and their assembly rules correlate with *ex silico* reactants and chemical reactions.

**Fig. 15:**
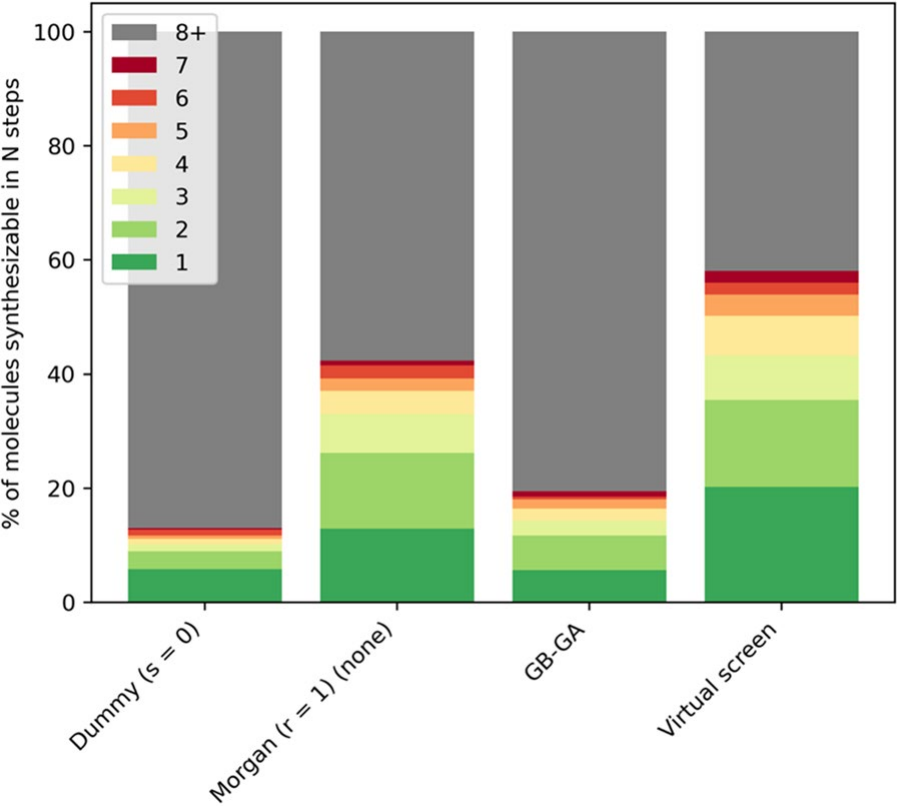
Fraction of top-10 scored molecules per replica synthesizable by LEADD (with different settings), GB-GA and VS in *N* or less steps using ZINC reactants and USPTO reaction templates, as assessed by AiZynthFinder. Molecules requiring more than 8 synthetic steps are considered not synthesizable

When using Morgan (r = 1) atom types and monolithic acyclic fragments LEADD designs molecules with much better SA than GB-GA (Fig. 14, Additional file 1: Table S12). This is to be expected since GB-GA doesn’t take SA into account intrinsically. However, it’s possible to design SA molecules with GB-GA by using the previously discussed extrinsic SAScore-based heuristic score modifier [14, 23]. Doing so yields a similar OP-SA trade-off to the one observed for LEADD and the same heuristic (Figs. 12, 13), strongly favoring SA over OP (Additional file 1: Figs. S7, S8). The SA of molecules designed by LEADD using Morgan (r = 1) atom types is almost on par with those found by a VS (Fig. 14, Additional file 1: Table S12). We would like to remark that the feature set we used to calculate SAScores was extracted from ChEMBL [50], and that the screened GuacaMol library is a subset of ChEMBL [13]. It’s therefore to be expected that molecules found through VS have better SAScores. Since SAScores are a rather crude way of assessing SA, to confirm our findings we ran retrosynthetic analyses on the top 10 scoring molecules of each benchmark replica using AiZynthFinder [21] with the ZINC [1] reactant stock and USPTO-derived reaction template policy provided by the authors. Both LEADD and GB-GA designed less synthesizable molecules than those found by the VS, but when using Morgan atom types LEADD designed considerably more synthesizable molecules than GB-GA (Fig. 15). It’s worth noting that only 60% of the molecules selected by the VS from the ChEMBL subset were deemed synthesizable by the retrosynthetic analyses. If we assume that all molecules in ChEMBL are synthesizable this would suggest that we might be underestimating the SA of molecules, including those designed by the EAs.

**Fig. 16:**
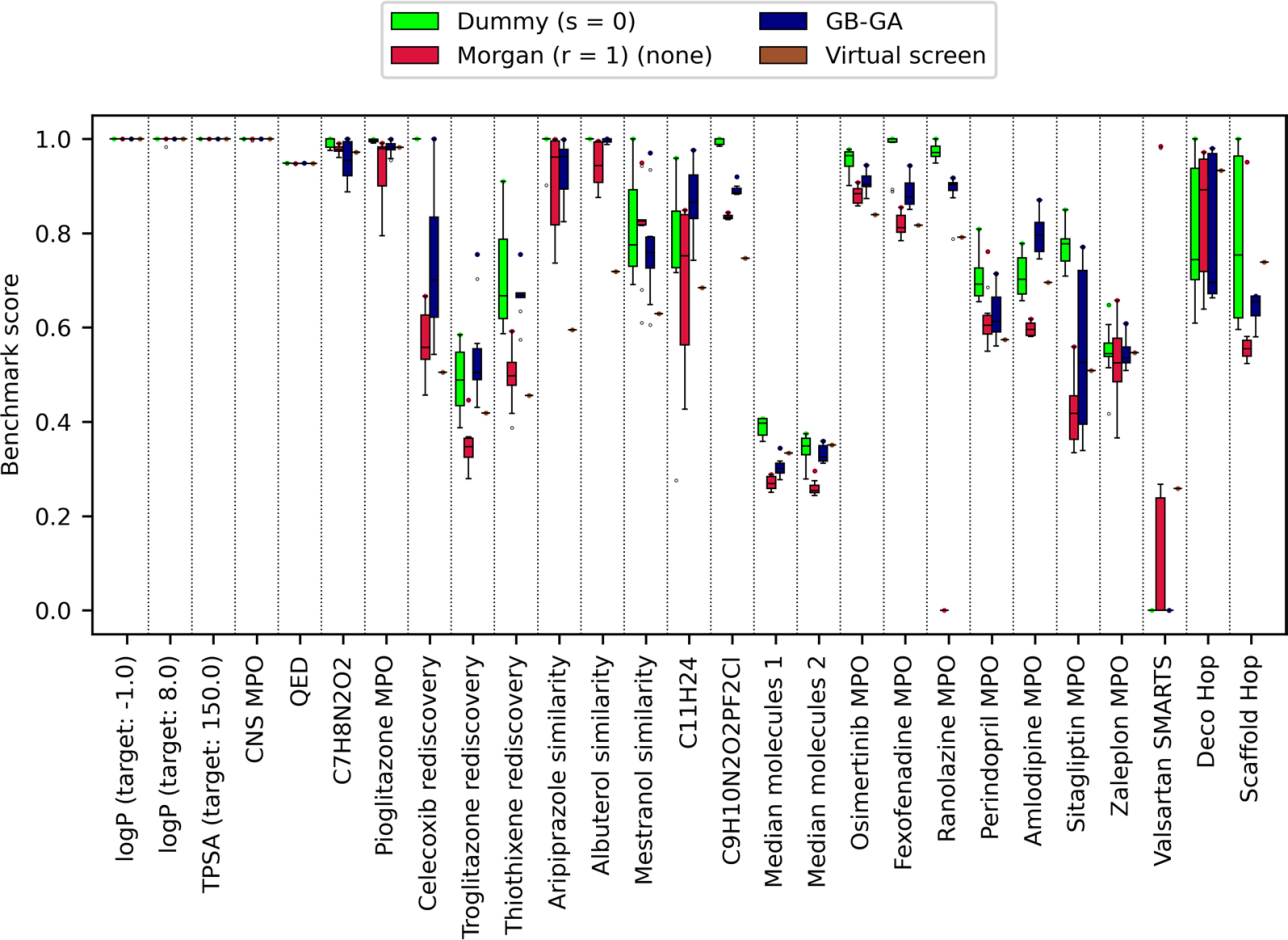
Optimization power comparison between LEADD, GB-GA and a VS. Benchmark scores range between 0 and 1, with higher scores being better. Boxes represent interquartile ranges (IQR), the black line within them medians and the whiskers Q ± 1.5IQR. Data beyond the whiskers are considered outliers and represented as dots. Colored dots represent maximum benchmark scores. Note that VS results are deterministic and have null variability

Interestingly, we didn’t observe a statistically significant difference in OP stochastic dominance between LEADD with Morgan atom types and GB-GA (Additional file 1: Table S13). Given that EAs are stochastic in nature, one would typically run multiple replicas and keep the best results. This justifies comparing maximum instead of average benchmark scores. In terms of maximum score, LEADD with Morgan atom types performed comparably or better than GB-GA in 16/27 benchmarks and comparably or better than the VS in 23/27 benchmarks (Fig. 16). Crucially, LEADD performed better than GB-GA in the Deco Hop and Scaffold Hop benchmarks, which are arguably the most representative of real drug discovery problems.

We would like to note that goal-directed design employing structure-based scoring functions is associated with an additional set of challenges that isn’t posed by the ligand-based GuacaMol benchmark suite, including the handling of stereochemistry, pose inversion and the typical bias of these scoring functions towards large, hydrophobic and flexible molecules. Indeed, preliminary results using OpenEye ROCS [57, 58] as LEADD’s scoring function show a tendency towards designing large and very cyclic molecules. This also makes it challenging to compare 2D molecular design algorithms like LEADD and GB-GA to their 3D counterparts [15, 22, 24, 39].

**Fig. 17:**
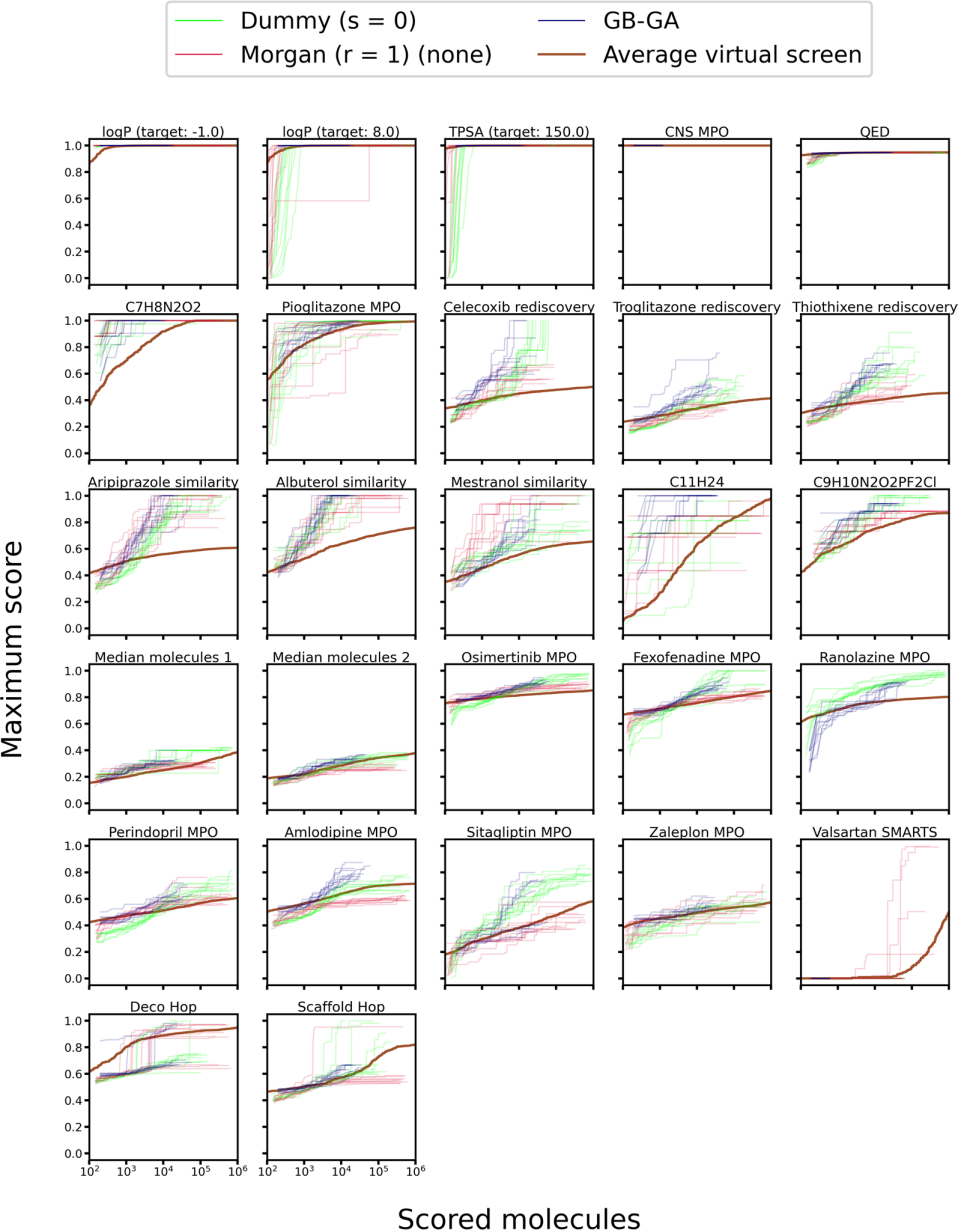
Score of best found molecule as a function of the number of scored molecules. For LEADD and GB-GA each line represents a replica. VS results were shuffled 100 times and averaged to account for the effects of molecule screening order. Note that these are individual molecule scores and not population/benchmark scores and therefore don’t correspond to the values in Fig. 16.

It’s also important to consider the amount of computational resources spent by each approach to achieve its results. Figure 17 shows how an average EA replica finds higher scoring molecules than a VS with a smaller number of scoring function calls. While one should keep in mind that it’s generally desirable to run multiple EA replicas, EAs make better use of computational resources than a VS, especially if evaluating the scoring function is expensive. It’s also worth noting that LEADD, despite its use of fragments, didn’t find solutions much slower than GB-GA (Fig. 17). Naturally, there is an overhead associated with the design algorithm. On a single core of a Xeon E5-2680v2 CPU (2.8 GHz), LEADD designed on average 272 mol/s. Assuming that about 10^4^–10^5^ molecules must be designed to find good solutions (Fig. 17) this corresponds to an overhead of just a couple CPU minutes. For comparison GB-GA designed 98 mol/s. This difference in performance is mostly due to implementation optimizations rather than due to algorithmic differences since LEADD is considerably more complex algorithmically. When using fast scoring functions molecule generation can become the rate limiting step. During the GuacaMol benchmark suite LEADD generated molecules slower than they were scored in 25/27 benchmarks. On average, molecules were designed eightfold slower than they were scored, with differences exceeding 20-fold in some benchmarks. This showcases the need for fast molecular design algorithms. Note that the reported values are averages, and that execution times depend heavily on the number of possibilities the algorithm has to consider. For instance, when using a smaller database of fragments or smaller population the algorithm is faster. Similarly, the computational resources spent per operation increase with molecular complexity, specifically degree of branching.

If one wishes to achieve even greater OP it’s possible to use the results of a VS as the starting population for EAs. While we don’t believe this qualifies as de novo molecular design, this type of molecular optimization may be interesting when computational resources are abundant. Unsurprisingly, we found that using VS results as starting populations decreased the variability between replicas and increased the mean replica score (Additional file 1: Fig. S9). However, when using Morgan atom types this didn’t always translate into higher maximum scores, as the starting population may already be close to local fitness minima in which the algorithm might get stuck. It’s interesting to note that, while the molecules designed this way have better SA than those in a true de novo design setting, it’s worse than that of the starting population (Additional file 1: Fig. S10). The SA loss is small for LEADD with Morgan atom types, but substantial for GB-GA and LEADD with dummy atom types, in the latter case almost reverting to the de novo design values. This showcases a tendency to design synthetically complex molecules when algorithms form bonds carelessly.

### Meaning of synthetic accessibility

Something all SA assessment tools have in common is that their judgements aren’t absolute but rather relative to the current chemical state of the art. Many DNDD algorithms, including LEADD, try to optimize the values of these SA predictions. One of the main appeals of DNDD is that it can suggest novel molecules. But if we constrict our search to the known types of chemistry that boast good SA predictions, how novel will these molecules truly be? Could our favor of the familiar lead us to neglect large areas of (potentially interesting) chemical space? Given the large uncertainty surrounding molecular activity predictions focusing research efforts on familiar chemistry is a reasonable way of increasing a project’s chances of success, but at a larger scale we risk creating a self-perpetuating cycle that could lead to academic stagnation. If we are confident enough in the accuracy of our scoring functions perhaps we shouldn’t hold historic data in such high regard and occasionally venture into unknown territory.

## Conclusions

We describe a novel set of genetic operators for fragment- and graph-based evolutionary molecular design algorithms that can enforce an arbitrary set of atom compatibility rules in a computationally efficient manner. Here we applied these genetic operators to achieve an improvement in OP and SA of designed molecules compared to other EAs.

## Supplementary Information

**Additional file 1. ** Additional figures, tables and methodology, including fragment database information, LEADD settings and statistical test results.

## Data Availability

LEADD’s source code can be found on the project’s GitHub repository (https://github.com/UAMCAntwerpen/LEADD). Fragments and compatibility rules were extracted from the GuacaMol “all SMILES” (v1) dataset, available at the GuacaMol GitHub repository (https://github.com/BenevolentAI/guacamol).

## Abbreviations

VS: Virtual screen/virtual screening
SA: Synthetically accessible/synthetic accessibility
OP: Optimization power
EA: Evolutionary algorithm
DNDD: De novo drug design
SSSR: Smallest Set of Smallest Rings
CXSMILES: ChemAxon extended SMILES
MBPM: Maximum Bipartite Matching Problem
MSI: Multiple set intersection
ANOVA: Analysis of variance
FWER: Family-wise error rate
USPTO: United States Patent and Trademark Office
