# Supplementary material for: LEADD: Lamarckian evolutionary algorithm for de novo drug design
Source: J Cheminform. 2022 Jan 15;14:3. doi: 10.1186/s13321-022-00582-y (PMC8760751; doi:10.1186/s13321-022-00582-y)
Supplement: Supplementary file 1 — Additional file 1. Additional figures, tables and methodology, including fragment database information, LEADD settings and statistical test results. [file 13321_2022_582_MOESM1_ESM.docx]

Additional file:

“LEADD: Lamarckian Evolutionary Algorithm for *de novo* Drug Design”

Alan Kerstjens*, Hans De Winter*

*Department of Pharmaceutical Sciences, Faculty of Pharmaceutical, Biomedical and Veterinary Sciences, University of Antwerp (Universiteitsplein 1A, Wilrijk 2610, Belgium).


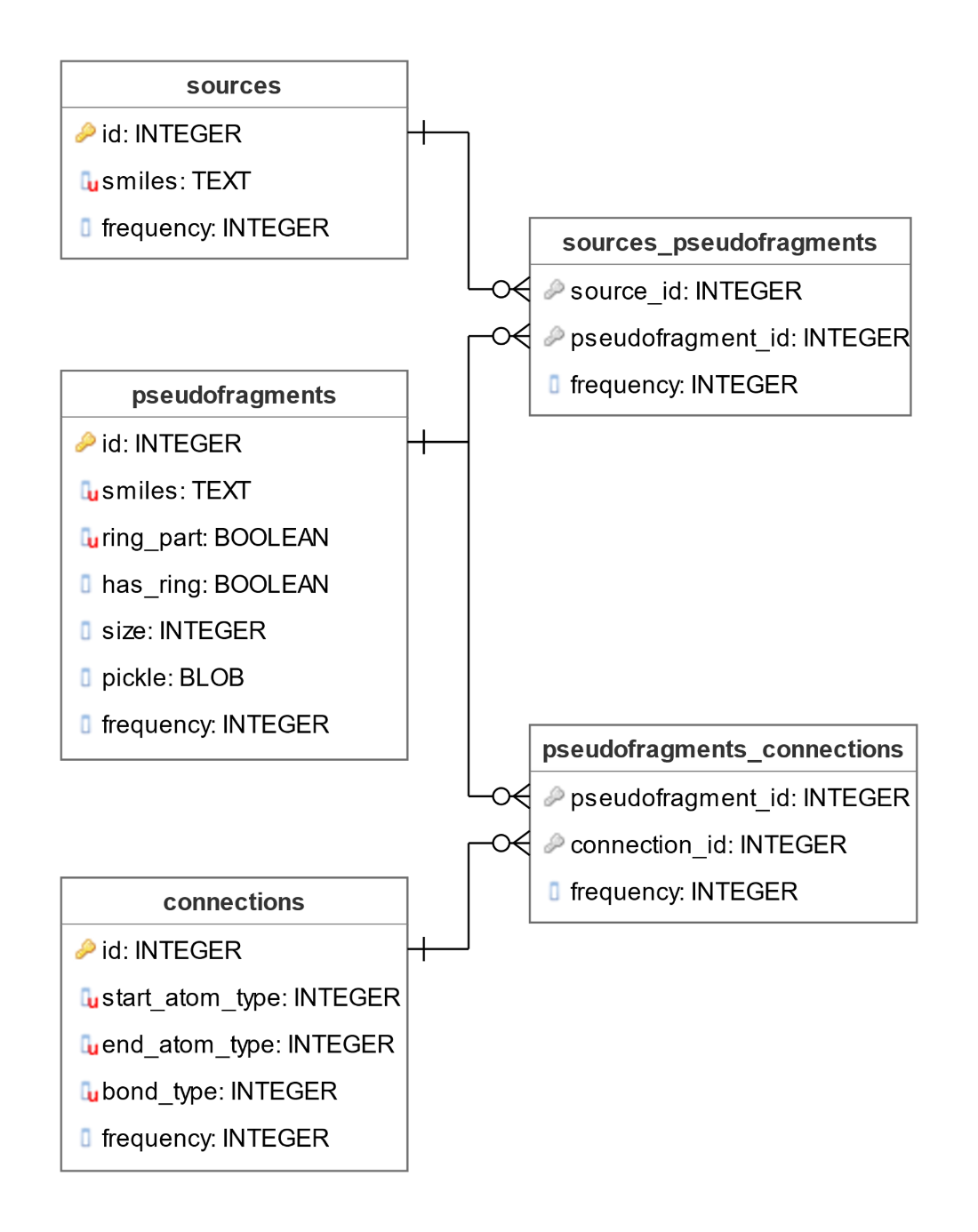


Figure S1. Schema of the relational SQLite3 fragments database, consisting of three main tables storing the molecules from which fragments were extracted (sources), the fragments themselves (pseudofragments) and the connections defined during fragmentation (connections). Two bridging tables express the relationships between the main tables.


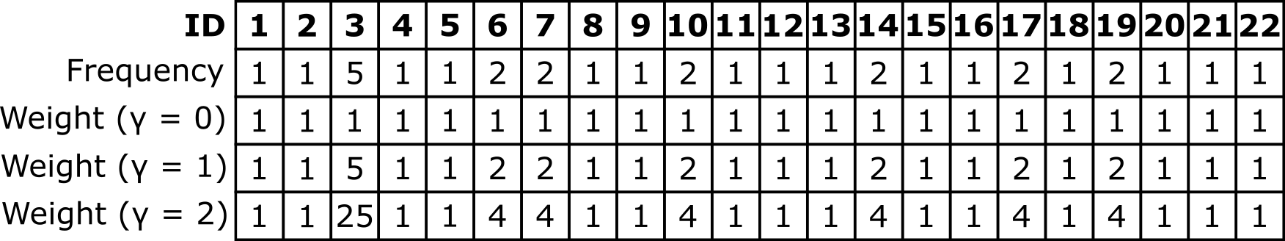


Figure S2. Example of how the weights of the fragments in Figure 1 are calculated according to their database frequency and the γ exponent (λ = 0) (Equation 1). Note that in practice a single γ is chosen.

Table S1. Fragment database and connection compatibility statistics for the explored combinations of atom typing scheme, fragmentation scheme and MBPM compatibility stringency. ^a^ For dummy atom types the strict and lax compatibility definitions are equivalent since only one atom type exists. ^b^ According to the compatibility definition stringency used for MBPM (column 3).

| **Atom typing scheme** | **Acyclic region fragmentation**  **scheme** | **MBPM compatibility stringency** | **Number of unique…** | | | | | **Average number of compatible…** | | | |
| --- | --- | --- | --- | --- | --- | --- | --- | --- | --- | --- | --- |
|  |  |  | **atom types** | **connections** | **acyclic fragments** | **ring fragments** | **connections**  **/connection (MBPM^b^)** | | **fragments**  **/connection (strict)** | **fragments**  **/connection (MBPM^b^)** |  |
| **Dummy** | **Subgraph**  **(s = 0)** | **Strict/Lax^a^** | 1 | 3 | 116 | 73287 | 1 | | 37417 | 37417 |  |
| **Dummy** | **Subgraph**  **(s ϵ [0 .. 2])** | **Strict/Lax^a^** | 1 | 3 | 3834 | 73287 | 1 | | 39286.7 | 39286.7 |  |
| **Dummy** | **None** | **Strict/Lax^a^** | 1 | 2 | 95430 | 73287 | 1 | | 104218 | 104218 |  |
| **MMFF** | **Subgraph**  **(s = 0)** | **Strict** | 64 | 1316 | 4869 | 205637 | 1 | | 436.8 | 436.8 |  |
| **MMFF** | **Subgraph**  **(s = 0)** | **Lax** | 64 | 1316 | 4869 | 205637 | 708.2 | | 436.8 | 180955 |  |
| **Morgan**  **(r = 1)** | **Subgraph**  **(s = 0)** | **Lax** | 14811 | 130494 | 139774 | 522517 | 1157.7 | | 14.1 | 10127.3 |  |
| **Morgan (r = 1)** | **Subgraph**  **(s = 0)** | **Strict** | 14811 | 130494 | 139774 | 522517 | 1 | | 14.1 | 14.1 |  |
| **Morgan**  **(r = 1)** | **Subgraph**  **(s ϵ [0 .. 2])** | **Lax** | 14811 | 130494 | 937087 | 522517 | 1157.7 | | 31.7 | 39008 |  |
| **Morgan**  **(r = 1)** | **None** | **Lax** | 10472 | 62021 | 243964 | 522517 | 591.5 | | 30.7 | 16367.3 |  |
| **Morgan**  **(r = 2)** | **Subgraph**  **(s = 0)** | **Lax** | 381252 | 1334292 | 799676 | 942568 | 223.9 | | 3.2 | 845.2 |  |

## Cyclicity control

How a genetic operator will modulate *N_r_* is based on a pseudorandom number generator and the probabilities returned by up to two functions operating in tandem. The first function returns the probability of keeping the number of rings constant (*P^=^*) based on the current *N_r_*. It consists of a discrete function fit to the shape of a normal distribution’s probability density function (*PDF*), and with its maximum scaled to an arbitrary user provided value (*M*). The equations of the normal distribution’s PDF and discrete function are given in Equation S1 and Equation S2 respectively.

Equation S1

$$PDF\left( N_{r} \right)=\frac{1}{\sigma\sqrt{2\pi}}\cdot e^{-\frac{1}{2}\left( \frac{N_{r}-\mu}{\sigma} \right)^{2}}$$

Equation S2

$$P^{=}\left( N_{r} \right)=\frac{PDF(N_{r})}{\sum_{N_{r}=0}^{Max(N_{r})} PDF(N_{r})}\cdot\frac{M}{PDF(\mu)}$$

Equation S3

$$P^{\neq}\left( N_{r} \right)=1-P^{=}(N_{r})$$

The mean of the normal distribution (*μ*) describes the ideal *N_r_* and its standard deviation (*σ*) the leniency in oscillating said number during evolution. Both parameters are user provided and ideally based on some notion of the desired *N_r_* in a solution. Both *PDF(N_r_)* and *P^=^* are maximum at *μ* and equal to the user’s scaling target value *M*.

While for expansions and deletions this function suffices to decide how to modulate the number of rings, for substitutions and transfections, if in the preceding step it was decided to change *N_r_*, a second function returns the probability of increasing it (*P^+^*) (Equation S4). This function is a logistic function defined based on *μ* and *σ*.

Equation S4

$$P^{+}\left( N_{r} \right)=P^{\neq}(N_{r})\cdot\frac{1}{1+e^{0.341\sigma\cdot(N_{r}-\mu)}}$$

Equation S5

$$P^{-}(N_{r})=P^{\neq}\left( N_{r} \right)-P^{+}\left( N_{r} \right)$$

The growth rate of the logistic function is set to be approximately the same as the normal distribution’s “steepness”, namely *0.682/2σ*, since in a normal distribution 68,2% of values are in *[μ – σ, μ + σ]*. Additionally, by setting the midpoint of the logistic function to *μ*, *P^+^(μ) = P^-^(μ)*. The special case where *N_r_ = 0* is treated by setting *P^-^ = 0*.

An example of the three aforementioned probability curves is shown in Figure S3.


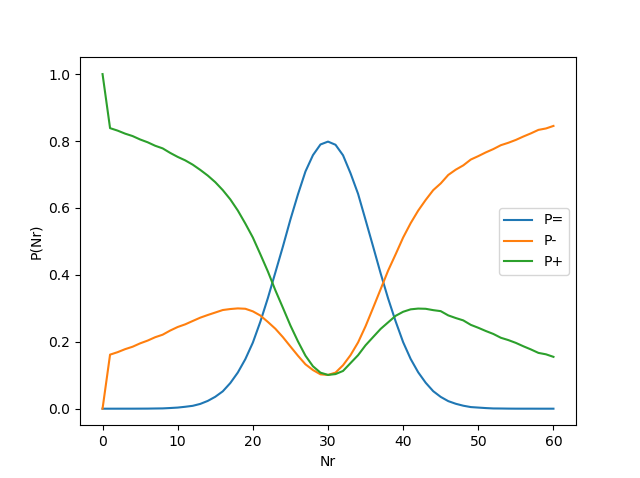


Figure S3. Probability distributions of keeping the number of ring atoms (N_r_) constant (P^=^), increasing it (P^+^) or decreasing it (P^-^) based on the current N_r_, as described by Equation S1 - Equation S5. μ = 30, σ = 6, M = 0.8.


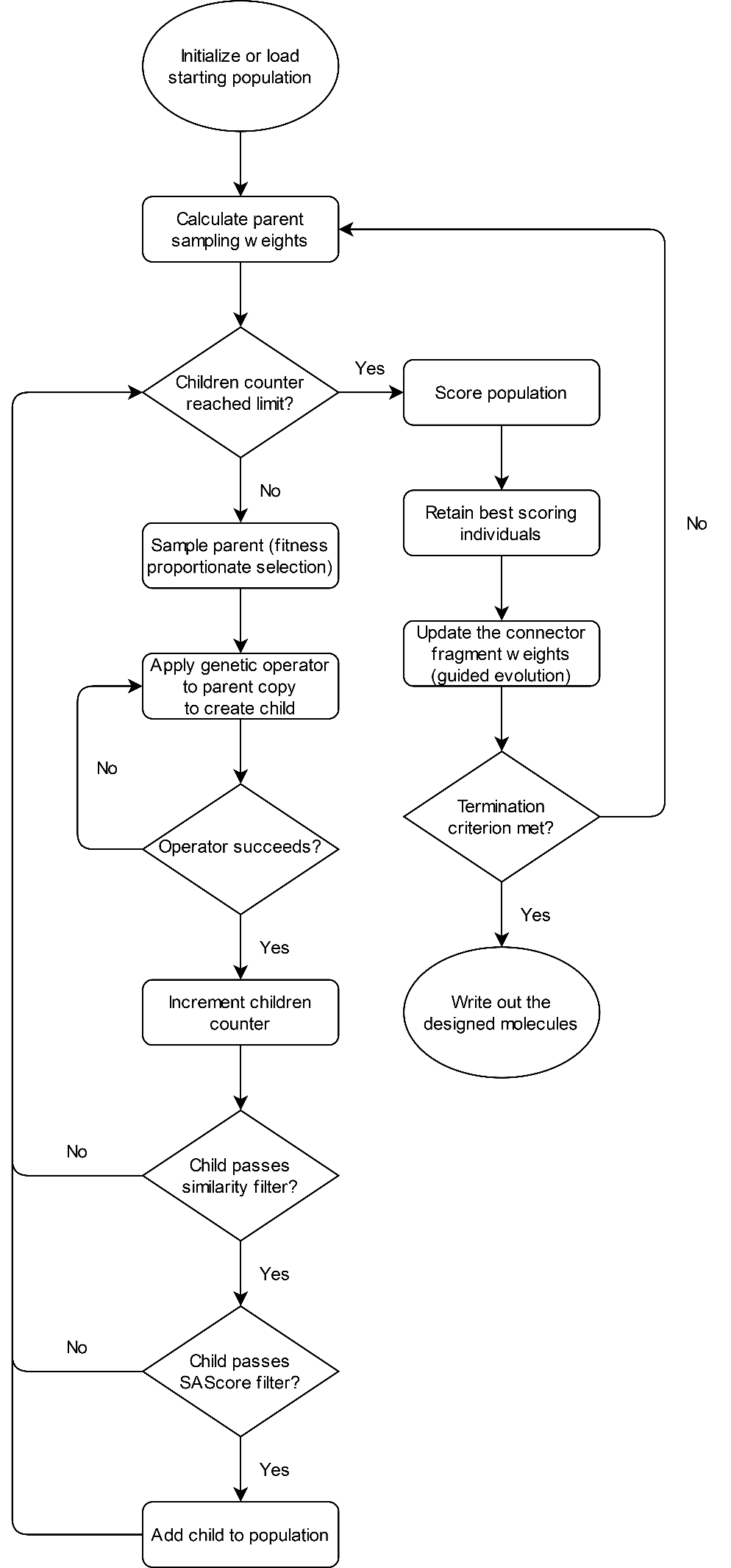


Figure S4. Flowchart of LEADD’s main loop. Note that some of the flowchart’s steps are optional, including the internal similarity and SAScore filters and the guided evolution.

Table S2. LEADD’s cyclicity control settings used during the GuacaMol benchmark. N_r_ stands for number of ring atoms.

| **GuacaMol benchmark suite** | **Benchmark name** | **Mean N_r_** | **Standard deviation N_r_** | **Maximum N_r_** |
| --- | --- | --- | --- | --- |
| Trivial | logP (target: -1.0) | 6 | 3 | 18 |
|  | logP (target: 8.0) | 12 | 6 | 36 |
|  | TPSA (target: 150.0) | 18 | 9 | 54 |
|  | CNS MPO | 6 | 3 | 18 |
|  | QED | 18 | 9 | 54 |
|  | C7H8N2O2 | 6 | 3 | 18 |
|  | Pioglitazone MPO | 17 | 8.5 | 51 |
| V2 | Celecoxib rediscovery | 17 | 8.5 | 51 |
|  | Troglitazone rediscovery | 21 | 10.5 | 63 |
|  | Thiothixene rediscovery | 20 | 10 | 60 |
|  | Aripiprazole similarity | 22 | 11 | 66 |
|  | Albuterol similarity | 6 | 3 | 18 |
|  | Mestranol similarity | 17 | 8.5 | 51 |
|  | C11H24 | 6 | 3 | 18 |
|  | C9H10N2O2PF2Cl | 6 | 3 | 18 |
|  | Median molecules 1 | 6 | 3 | 18 |
|  | Median molecules 2 | 23 | 11.5 | 69 |
|  | Osimertinib MPO | 21 | 10.5 | 63 |
|  | Fexofenadine MPO | 24 | 12 | 72 |
|  | Ranolazine MPO | 18 | 9 | 54 |
|  | Perindopril MPO | 9 | 4.5 | 27 |
|  | Amlodipine MPO | 12 | 6 | 36 |
|  | Sitagliptin MPO | 15 | 7.5 | 45 |
|  | Zaleplon MPO | 15 | 7.5 | 45 |
|  | Valsartan SMARTS | 17 | 8.5 | 51 |
|  | Deco Hop | 20 | 10 | 60 |
|  | Scaffold Hop | 20 | 10 | 60 |

Table S3. Summary of LEADD’s default reconstruction settings. Some settings were condensed or omitted from this table. For a more detailed list of settings we refer readers to the source code.

| **Parameter name** | **True *de novo* design value** |
| --- | --- |
| Fragment frequency exponent (*γ*) | 1.0 |
| Fragment size exponent (*λ*) | 0.0 |
| Molecule score exponent (*ζ*) | 2.5 |
| Peripheral expansion weight | 4.0 |
| Internal expansion weight | 4.0 |
| Peripheral deletion weight | 4.0 |
| Internal deletion weight | 4.0 |
| Substitution weight | 56.0 |
| Transfection weight | 20.0 |
| Translation weight | 4.0 |
| Stereo-flip weight | 0.0 |
| Randomize unspecified stereo | False |
| # seed molecules | 100 |
| # children per generation | 100 |
| # survivors per generation | 100 |
| Maximum child similarity | 0.9 |
| Maximum # generations | 10,000 |
| Maximum # generations stuck | 1,000 |
| SAScore filter | Disabled |
| SAScore heuristic | Disabled |
| Lamarckian evolution guidance | Disabled |

Table S4. Multiple comparisons of SAScore means using different atom typing schemes with Tukey’s HSD post-hoc test (FWER = 0.05). The test was preceded by a one-way ANOVA (F = 5675.82, p < 0.001).

| **Group 1** | **Group 2** | ${\bar{\boldsymbol{SAScore}}}_{\boldsymbol{Group}\boldsymbol{2}}\boldsymbol{-}{\bar{\boldsymbol{SAScore}}}_{\boldsymbol{Group}\boldsymbol{1}}$ | **Adjusted p-value** |
| --- | --- | --- | --- |
| Dummy | MMFF | 0.0636 | < 0.001 |
| Dummy | Morgan (r = 1) | -0.7070 | < 0.001 |
| Dummy | Morgan (r = 2) | -0.817 | < 0.001 |
| MMFF | Morgan (r = 1) | -0.7706 | < 0.001 |
| MMFF | Morgan (r = 2) | -0.8806 | < 0.001 |
| Morgan (r = 1) | Morgan (r = 2) | -0.1101 | < 0.001 |

Table S5. Multiple comparisons of benchmark score distributions´ stochastic dominances using different atom typing schemes with Conover-Iman´s post-hoc test with Šidák correction (FWER = 0.05). The test was preceded by a Kruskal-Wallis test (H = 149.90, p < 0.001).

| **Group 1** | **Group 2** | ${\tilde{\boldsymbol{Score}}}_{\boldsymbol{Group}\boldsymbol{2}}\boldsymbol{-}{\tilde{\boldsymbol{Score}}}_{\boldsymbol{Group}\boldsymbol{1}}$ | **Adjusted p-value** |
| --- | --- | --- | --- |
| Dummy | MMFF | -0.066 | 0.189 |
| Dummy | Morgan (r = 1) | -0.302 | < 0.001 |
| Dummy | Morgan (r = 2) | -0.384 | < 0.001 |
| MMFF | Morgan (r = 1) | -0.236 | < 0.001 |
| MMFF | Morgan (r = 2) | -0.318 | < 0.001 |
| Morgan (r = 1) | Morgan (r = 2) | -0.082 | 0.099 |


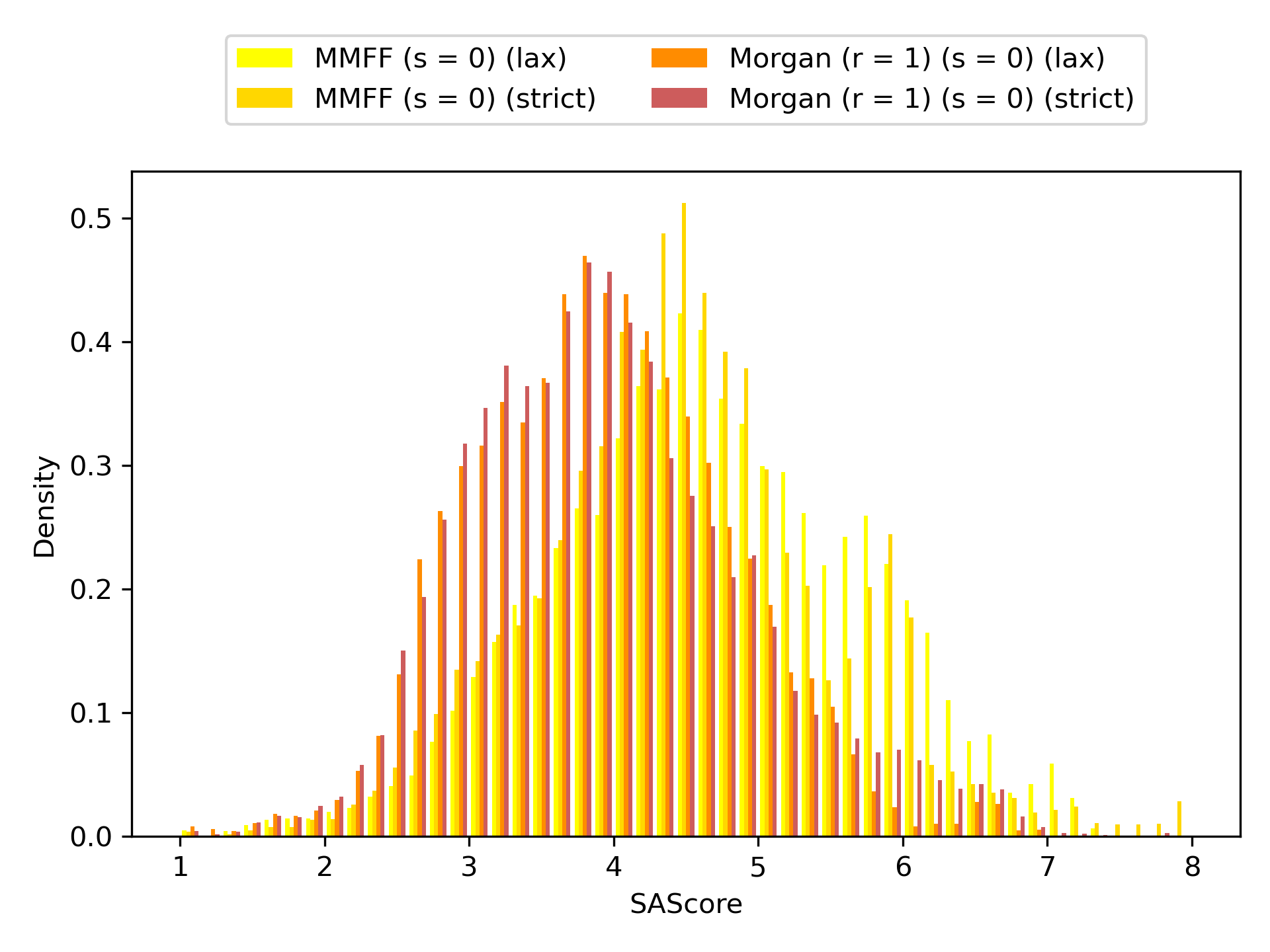


Figure S5. Comparison of designed molecules’ SAScore distributions using different MBPMB connection compatibility stringencies. Includes molecules of all benchmarks and replicas. Molecules with lower SAScores are predicted to be easier to synthesize.

Table S6. Two-way ANOVA on the effect of atom typing scheme, fragmentation scheme and their interaction on the SAScore of designed molecules.

| **Source of variation** | **df** | **Sum Sq** | **Mean Sq** | **F** | **p-value** |
| --- | --- | --- | --- | --- | --- |
| Atom typing | 1 | 15,205.18 | 15,205.18 | 16,055.82 | < 0.001 |
| Fragmentation | 2 | 1,879.06 | 939.53 | 992.09 | < 0.001 |
| Interaction | 2 | 119.08 | 59.54 | 62.87 | < 0.001 |
| Residual | 154,720 | 146,522.90 | 0.95 |  |  |

Table S7. Multiple comparisons of SAScore means using different combinations of atom typing and fragmentation schemes with Tukey’s HSD post-hoc test (FWER = 0.05).

| **Group 1** | **Group 2** | ${\bar{\boldsymbol{SAScore}}}_{\boldsymbol{Group}\boldsymbol{2}}\boldsymbol{-}{\bar{\boldsymbol{SAScore}}}_{\boldsymbol{Group}\boldsymbol{1}}$ | **Adjusted p-value** |
| --- | --- | --- | --- |
| Dummy (s = 0) | Dummy (s ϵ [0 .. 2]) | -0.1030 | < 0.001 |
| Dummy (s = 0) | Dummy (none) | -0.3111 | < 0.001 |
| Dummy (s = 0) | Morgan (r = 1) (s = 0) | -0.7070 | < 0.001 |
| Dummy (s = 0) | Morgan (r = 1) (s ϵ [0 .. 2]) | -0.6913 | < 0.001 |
| Dummy (s = 0) | Morgan (r = 1) (none) | -0.9016 | < 0.001 |
| Dummy (s ϵ [0 .. 2]) | Dummy (none) | -0.2082 | < 0.001 |
| Dummy (s ϵ [0 .. 2]) | Morgan (r = 1) (s = 0) | -0.6040 | < 0.001 |
| Dummy (s ϵ [0 .. 2]) | Morgan (r = 1) (s ϵ [0 .. 2]) | -0.5884 | < 0.001 |
| Dummy (s ϵ [0 .. 2]) | Morgan (r = 1) (none) | -0.7987 | < 0.001 |
| Dummy (none) | Morgan (r = 1) (s = 0) | -0.3959 | < 0.001 |
| Dummy (none) | Morgan (r = 1) (s ϵ [0 .. 2]) | -0.3802 | < 0.001 |
| Dummy (none) | Morgan (r = 1) (none) | -0.5905 | < 0.001 |
| Morgan (r = 1) (s = 0) | Morgan (r = 1) (s ϵ [0 .. 2]) | 0.0157 | 0.4443 |
| Morgan (r = 1) (s = 0) | Morgan (r = 1) (none) | -0.1946 | < 0.001 |
| Morgan (r = 1) (s ϵ [0 .. 2]) | Morgan (r = 1) (none) | -0.2103 | < 0.001 |

Table S8. Scheirer-Ray-Hare test on the effect of atom typing scheme, fragmentation scheme and their interaction on the GuacaMol benchmark scores.

| **Source of variation** | **df** | **Sum Sq** | **H** | **p-value** |
| --- | --- | --- | --- | --- |
| Atom typing | 1 | 36,511,969 | 169.401 | < 0.001 |
| Fragmentation | 2 | 1,854,912 | 8.606 | 0.014 |
| Interaction | 2 | 439,082 | 2.037 | 0.361 |
| Residual | 1,604 | 307,991,950 |  |  |

Table S9. Multiple comparisons of benchmark score distributions’ stochastic dominances using different combinations of atom typing and fragmentation schemes with Conover-Iman´s post-hoc test with Šidák correction (FWER = 0.05)

| **Group 1** | **Group 2** | ${\tilde{\boldsymbol{Score}}}_{\boldsymbol{Group}\boldsymbol{2}}\boldsymbol{-}{\tilde{\boldsymbol{Score}}}_{\boldsymbol{Group}\boldsymbol{1}}$ | **Adjusted p-value** |
| --- | --- | --- | --- |
| Dummy (s = 0) | Dummy (s ϵ [0 .. 2]) | 0.006 | 0.949 |
| Dummy (s = 0) | Dummy (none) | 0.012 | 0.900 |
| Dummy (s = 0) | Morgan (r = 1) (s = 0) | -0.302 | < 0.001 |
| Dummy (s = 0) | Morgan (r = 1) (s ϵ [0 .. 2]) | -0.176 | < 0.001 |
| Dummy (s = 0) | Morgan (r = 1) (none) | -0.122 | < 0.001 |
| Dummy (s ϵ [0 .. 2]) | Dummy (none) | 0.006 | 0.998 |
| Dummy (s ϵ [0 .. 2]) | Morgan (r = 1) (s = 0) | -0.308 | < 0.001 |
| Dummy (s ϵ [0 .. 2]) | Morgan (r = 1) (s ϵ [0 .. 2]) | -0.182 | < 0.001 |
| Dummy (s ϵ [0 .. 2]) | Morgan (r = 1) (none) | -0.128 | < 0.001 |
| Dummy (none) | Morgan (r = 1) (s = 0) | -0.315 | < 0.001 |
| Dummy (none) | Morgan (r = 1) (s ϵ [0 .. 2]) | -0.188 | < 0.001 |
| Dummy (none) | Morgan (r = 1) (none) | -0.134 | < 0.001 |
| Morgan (r = 1) (s = 0) | Morgan (r = 1) (s ϵ [0 .. 2]) | 0.127 | 0.185 |
| Morgan (r = 1) (s = 0) | Morgan (r = 1) (none) | 0.180 | 0.015 |
| Morgan (r = 1) (s ϵ [0 .. 2]) | Morgan (r = 1) (none) | 0.053 | 0.932 |

Table S10. Multiple comparisons of SAScore means using different approaches to improve SA with Tukey’s HSD post-hoc test (FWER = 0.05). The test was preceded by a one-way ANOVA (F = 45720.82, p < 0.001).

| **Group 1** | **Group 2** | ${\bar{\boldsymbol{SAScore}}}_{\boldsymbol{Group}\boldsymbol{2}}\boldsymbol{-}{\bar{\boldsymbol{SAScore}}}_{\boldsymbol{Group}\boldsymbol{1}}$ | **Adjusted p-value** |
| --- | --- | --- | --- |
| Dummy | Morgan (r = 1) | -0.9016 | < 0.001 |
| Dummy | Dummy  (SAScore filter) | -0.5988 | < 0.001 |
| Dummy | Dummy  (SAScore heuristic) | -2.3578 | < 0.001 |
| Morgan (r = 1) | Dummy  (SAScore filter) | 0.3028 | < 0.001 |
| Morgan (r = 1) | Dummy  (SAScore heuristic) | -1.4561 | < 0.001 |
| Dummy  (SAScore filter) | Dummy  (SAScore heuristic) | -1.7590 | < 0.001 |

Table S11. Multiple comparisons of benchmark score distributions’ stochastic dominances using different approaches to improve SA with Conover-Iman´s post-hoc test with Šidák correction (FWER = 0.05). The test was preceded by a Kruskal-Wallis test (H = 94.69, p < 0.001).

| **Group 1** | **Group 2** | ${\tilde{\boldsymbol{Score}}}_{\boldsymbol{Group}\boldsymbol{2}}\boldsymbol{-}{\tilde{\boldsymbol{Score}}}_{\boldsymbol{Group}\boldsymbol{1}}$ | **Adjusted p-value** |
| --- | --- | --- | --- |
| Dummy | Morgan (r = 1) | -0.122 | < 0.001 |
| Dummy | Dummy  (SAScore filter) | -0.027 | 0.724 |
| Dummy | Dummy  (SAScore heuristic) | -0.253 | < 0.001 |
| Morgan (r = 1) | Dummy  (SAScore filter) | 0.096 | < 0.001 |
| Morgan (r = 1) | Dummy  (SAScore heuristic) | -0.161 | 0.006 |
| Dummy  (SAScore filter) | Dummy  (SAScore heuristic) | -0.226 | < 0.001 |

##
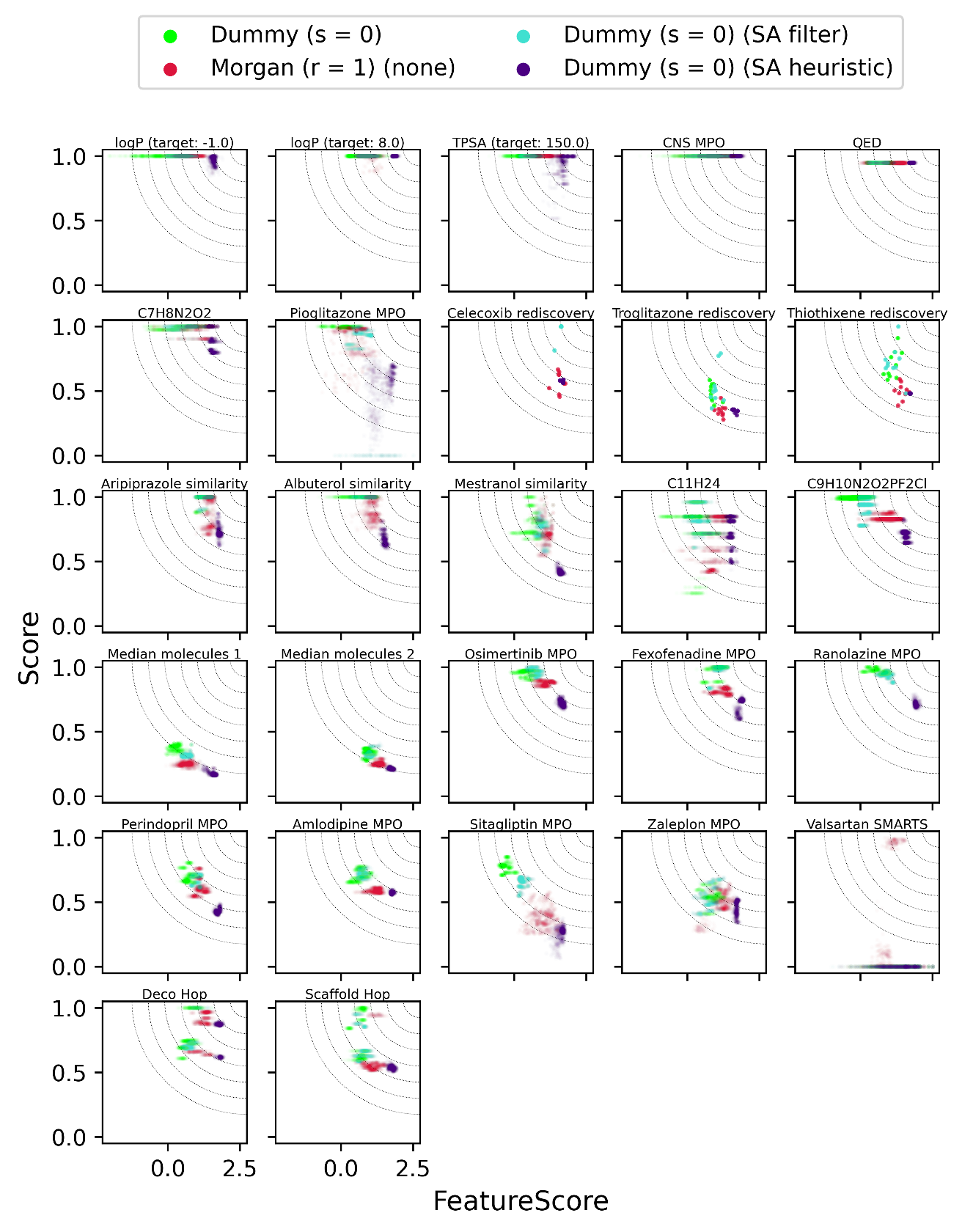


Figure S6. Correlation between FeatureScore and benchmark score using different approaches to increase SA. Large feature scores are associated with better SA.


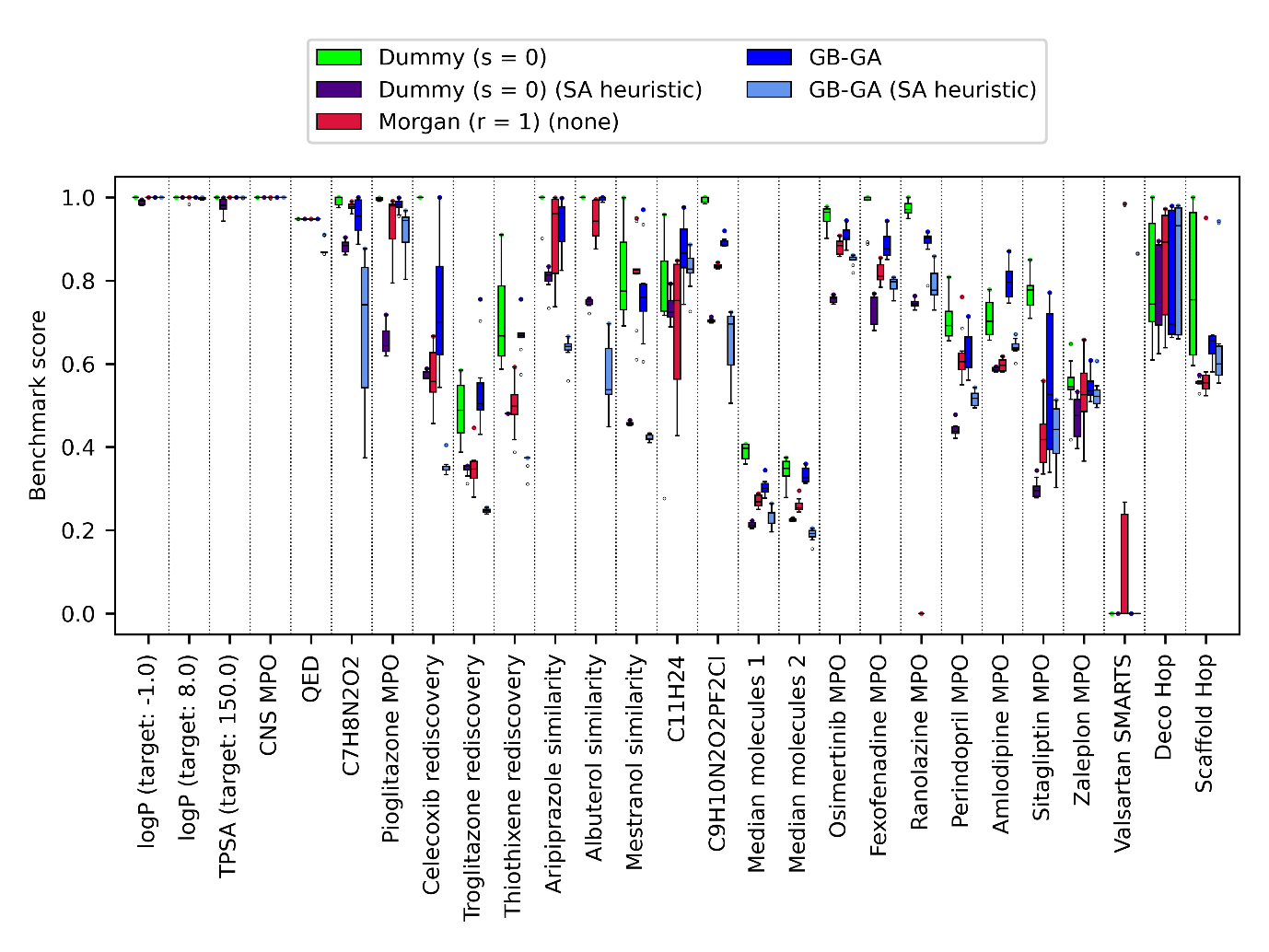


Figure S7. Optimization power comparison between LEADD and GB-GA with or without using the SAScore-based score modifier. Benchmark scores range between 0 and 1, with higher scores being better. Boxes represent interquartile ranges (IQR), the black line within them medians and the whiskers Q±1.5IQR. Data beyond the whiskers are considered outliers and represented as dots. Colored dots represent maximum benchmark scores.


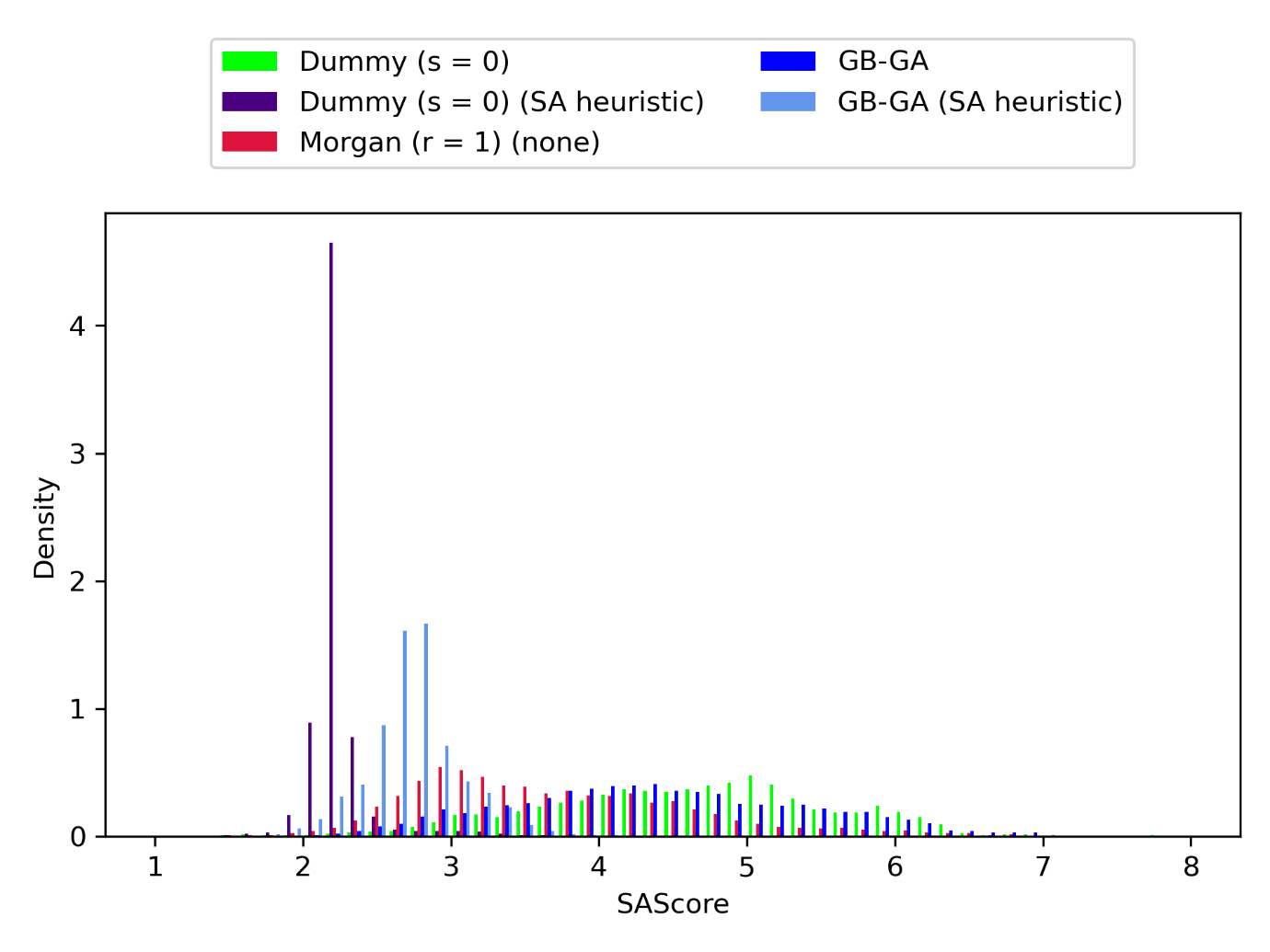


Figure S8. Comparison of SAScore distributions between molecules designed by LEADD and GB-GA with or without using the SAScore-based score modifier. Includes molecules of all benchmarks and replicas. Molecules with lower SAScores are predicted to be easier to synthesize.

Table S12. Multiple comparisons of SAScore means between LEADD, GB-GA and a VS with Tukey’s HSD post-hoc test (FWER = 0.05). The test was preceded by a one-way ANOVA (F = 5715.55, p < 0.001).

| **Group 1** | **Group 2** | ${\bar{\boldsymbol{SAScore}}}_{\boldsymbol{Group}\boldsymbol{2}}\boldsymbol{-}{\bar{\boldsymbol{SAScore}}}_{\boldsymbol{Group}\boldsymbol{1}}$ | **Adjusted p-value** |
| --- | --- | --- | --- |
| Dummy | Morgan (r = 1) | -0.902 | < 0.001 |
| Dummy | GB-GA | -0.222 | < 0.001 |
| Dummy | VS | -1.170 | < 0.001 |
| Morgan (r = 1) | GB-GA | 0.680 | < 0.001 |
| Morgan (r = 1) | VS | -0.268 | < 0.001 |
| GB-GA | VS | -0.948 | < 0.001 |

Table S13. Multiple comparisons of benchmark score distributions´ stochastic dominances between LEADD, GB-GA and a VS with Conover-Iman´s post-hoc test with Šidák correction (FWER = 0.05). The test was preceded by a Kruskal-Wallis test (H = 36.83, p < 0.001). ^a^ VS results are deterministic, whereas for the EAs 10 replicas were ran. Hence, the VS sample size is 1/10^th^ that of the other groups, explaining the small statistical power and inflated p-values.

| **Group 1** | **Group 2** | ${\tilde{\boldsymbol{Score}}}_{\boldsymbol{Group}\boldsymbol{2}}\boldsymbol{-}{\tilde{\boldsymbol{Score}}}_{\boldsymbol{Group}\boldsymbol{1}}$ | **Adjusted p-value** |
| --- | --- | --- | --- |
| Dummy | Morgan (r = 1) | -0.122 | < 0.001 |
| Dummy | GB-GA | -0.076 | 0.001 |
| Dummy | VS | -0.230 | 0.024^a^ |
| Morgan (r = 1) | GB-GA | 0.046 | 0.137 |
| Morgan (r = 1) | VS | -0.108 | 1.000^a^ |
| GB-GA | VS | -0.154 | 0.723^a^ |

##
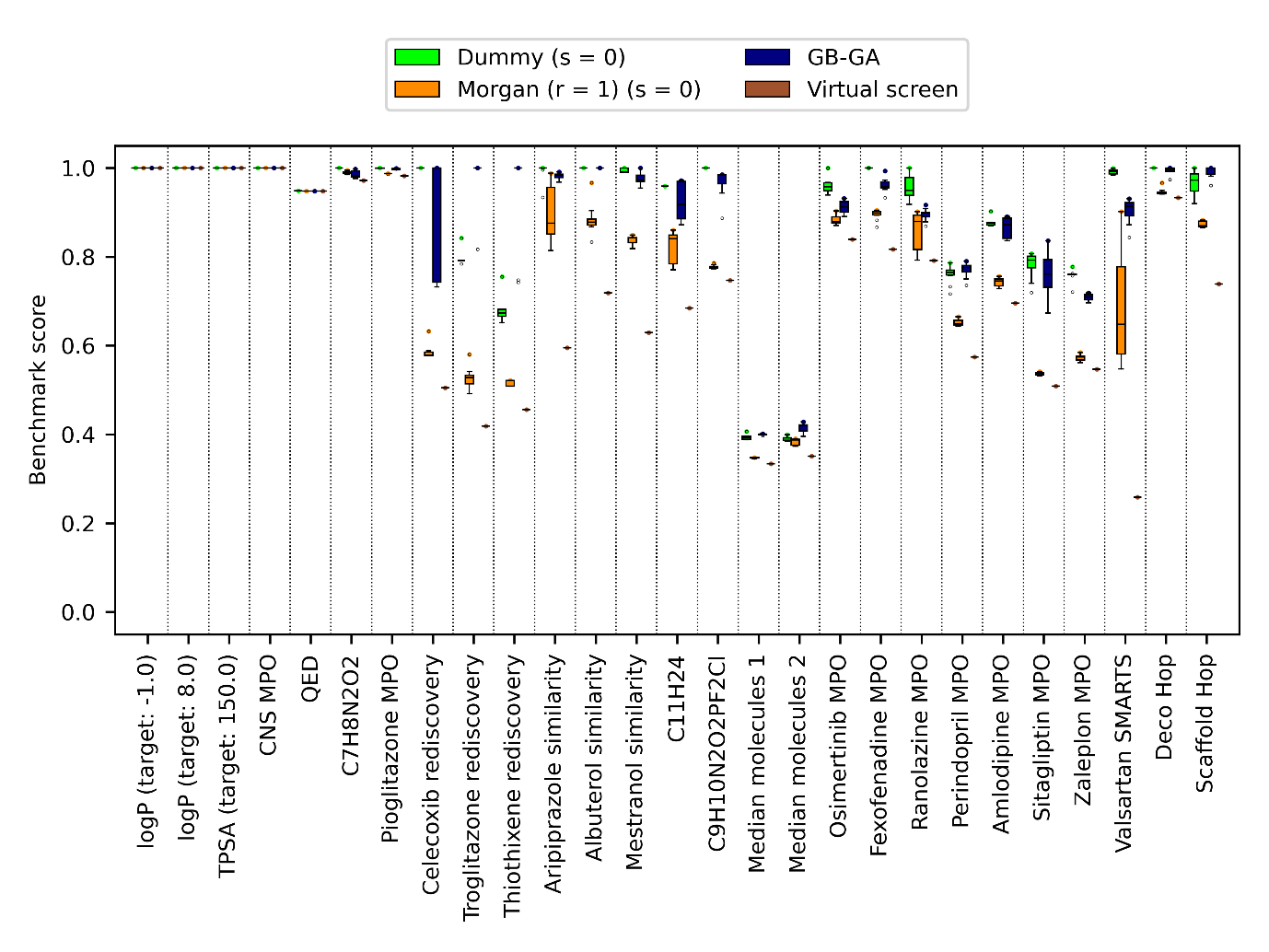


Figure S9. Optimization power comparison between LEADD, GB-GA and a VS using the VS results as starting populations for the EAs. Benchmark scores range between 0 and 1, with higher scores being better. Boxes represent interquartile ranges (IQR), the black line within them medians and the whiskers Q±1.5IQR. Data beyond the whiskers are considered outliers and represented as dots. Colored dots represent maximum benchmark scores. Note that VS results are deterministic and have null variability.


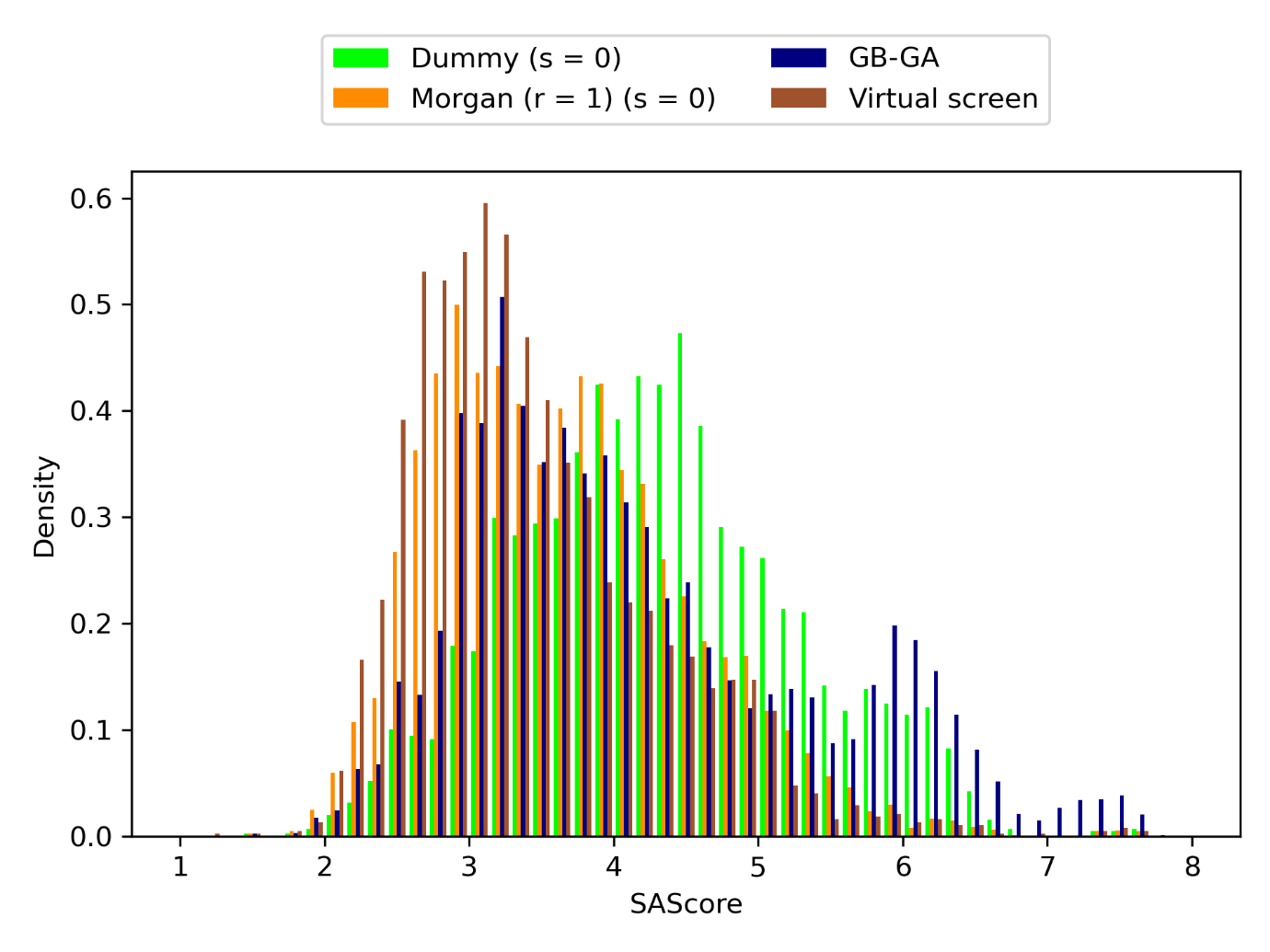


Figure S10. Comparison of SAScore distributions between molecules designed by LEADD and GB-GA using VS results as a starting population and said VS results. Includes molecules of all benchmarks and replicas. Molecules with lower SAScores are predicted to be easier to synthesize.
